# pyQms enables universal and accurate quantification of mass spectrometry data*[Fn FN2]

**DOI:** 10.1074/mcp.M117.068007

**Published:** 2017-07-20

**Authors:** Johannes Leufken, Anna Niehues, L. Peter Sarin, Florian Wessel, Michael Hippler, Sebastian A. Leidel, Christian Fufezan

**Affiliations:** From the ‡Institute of Plant Biology and Biotechnology, University of Muenster, Schlossplatz 8, 48143 Muenster, Germany;; §Max Planck Research Group for RNA Biology, Max Planck Institute for Molecular Biomedicine, Von-Esmarch-Strasse 54, 48149 Muenster, Germany;; ¶Deutsches Krebsforschungszentrum, G181 DKFZ-Bayer Joint Immunotherapy Laboratory, 69120 Heidelberg, Germany;; ‖Cells-in-Motion Cluster of Excellence, University of Muenster, 48149 Muenster, Germany;; **Faculty of Medicine, University of Muenster, Albert-Schweitzer-Campus 1, 48149 Muenster, Germany;; ‡‡Cellzome A GSK Company, Meyerhofstrasse 1, 69117 Heidelberg, Germany

## Abstract

Quantitative mass spectrometry (MS) is a key technique in many research areas (1), including proteomics, metabolomics, glycomics, and lipidomics. Because all of the corresponding molecules can be described by chemical formulas, universal quantification tools are highly desirable. Here, we present pyQms, an open-source software for accurate quantification of all types of molecules measurable by MS. pyQms uses isotope pattern matching that offers an accurate quality assessment of all quantifications and the ability to directly incorporate mass spectrometer accuracy. pyQms is, due to its universal design, applicable to every research field, labeling strategy, and acquisition technique. This opens ultimate flexibility for researchers to design experiments employing innovative and hitherto unexplored labeling strategies. Importantly, pyQms performs very well to accurately quantify partially labeled proteomes in large scale and high throughput, the most challenging task for a quantification algorithm.

Current mass spectrometric workflows use a plethora of labeling strategies (Fig. 1). Established examples are label-free quantification (Fig. 1*A*) and metabolic labeling with partially or fully enriched isotopes (Figs. 1*B* and 1*C*). Furthermore, labeled molecules can be added to the culture, as in stable isotope labeling with amino acids in cell culture (2) or can be introduced *in vitro* by chemical tagging, *e.g.* TMT10 (3, 4) (Fig. 1*D*). For certain research areas and labeling strategies, powerful data analysis tools are well established (5–14). However, existing software solutions are generally not universal as they have been tailored to specific research fields and are often restricted to defined experimental protocols (see Supplemental Table S1 for a summary). For example, some software can quantify peptides with artificial isotope distributions, but not metabolites, or can quantify molecules when they are metabolically labeled with nitrogen isotope ^15^N, but not if labeled with carbon isotope ^13^C. Finally, some labeling strategies can currently not be combined within the same experiment (Figs. 1*E* and 1*F*).

These limitations, however, are artificial since all quantified entities are molecules defined by chemical formulas with known isotope distributions and masses. pyQms takes advantage of this knowledge and treats all molecules as formulas to calculate accurate isotope patterns that are based on the labeling strategy. This liberates the algorithm to perform analyses irrespective of the type of molecule (protein, metabolite, lipid, glycan, etc.), the type of label (metabolic or fixed) or MS level. Accordingly, there is no restriction in quantifying any combination of labels within the same experiment, paving the way for innovative experimental designs that would be precluded with most quantification tools. pyQms has been evaluated for accuracy and sensitivity in label-free proteomics (Supplemental Figs. 1 *A*–1*C*) and for pulse (chase) metabolic labeling data analysis using a novel partially labeled proteome gold standard data set (Supplemental Figs. 1 *D*–1*J*).

## EXPERIMENTAL PROCEDURES

### 

#### 

##### Partially Labeled Proteome Gold Standard Data Set Sample Preparation

*Chlamydomonas reinhardtii* (strain CW15) cells were grown in photoheterotrophic conditions in tris-acetate-phosphate (TAP) medium (15) at a light intensity of 50 μE m^−2^ s^−1^ at 22 °C on a rotary shaker at 120 rpm or on TAP-agar plates containing 1.5% agar at a light intensity of 40–50 μE m^−2^ s^−1^. Metabolic labeling was performed by mixing unlabeled TAP medium with fully labeled TAP medium containing 100% ^15^N at different proportions (0, 20, 40, 60, 80, and 100% (w/w)). Fully labeled medium was created using 99.4% ^15^N enriched ^15^NH_4_Cl (Cambridge Isotope Laboratories, Tewksbury, MA). Cells were grown several generations on ^15^N containing agar plates to ensure complete metabolic labeling with the defined ^15^N proportion. Cells were then grown for 3 days in liquid medium, maintaining the labeling proportion and harvested at 5000 × *g* (Beckmann Coulter J 20 XP), suspended in H6 buffer (5 mm HEPES, pH 7.5, 10 mm EDTA), and stored at −80 °C. Protein samples were digested with trypsin using a modified filter-aided sample preparation protocol (16) as described in Barth *et al.* (17) with the following modifications: Samples were mixed based on equal chlorophyll content (6.25 μg) and washing steps were repeated four times.

##### LC-MS/MS Measurement

Liquid chromatography coupled tandem mass spectrometry (LC-MS/MS) measurements were done employing a Dionex Ultimate 3000 UPLC system (Thermo Scientific) and Q Exactive Plus (Thermo Scientific, Bremen, Germany) instrument. Software versions, which were used are: Exactive Series (Tune) 2.3 Build 1765 and Xcalibur 3.0.63. Peptides were separated by reversed phase chromatography. Peptide samples were loaded on a trap column (Acclaim PepMap100, 300 μm × 5 mm, 5 μm particle size, 100 Å pore size; Thermo Scientific, Bremen, Germany). Samples were desalted utilizing a flow rate of 5 μl/min for 5 min using 2% (v/v) acetonitrile/0.05% (v/v) trifluoroacetic acid in ultrapure water. Peptide separation was done using a mobile phase composed of 0.1% (v/v) formic acid in ultrapure water (A) and 80% (v/v) acetonitrile/0.1% (v/v) formic acid in ultrapure water (B). The trap column was switched for peptide elution in-line with a C18 capillary column (Acclaim PepMap 100, 75 μm × 150 mm, 2 μm particle size, 100 Å pore size, Thermo Scientific, Bremen, Germany). The gradient used was: 2.5–35% B (90 min), 35–99% B (5 min), 99% B (5 min). Ions were generated by electrospray ionization. For full scans a resolution of 70,000 at *m/z* 200 was used (maximum injection time: 50 ms, automatic gain control target: 1e^6^, range: 400–1600 *m/z*). For each full scan, the 12 most abundant precursor ions (charge 2+ to 7+) were selected for fragmentation (MS^2^) by higher energy c-trap dissociation. For MS^2^ scans a resolution of 17,500 at *m/z* 200 was used (maximum injection time: 50 ms, AGC target: 5e^4^, underfill ratio: 1%). A dynamic exclusion of 60 s for fragmented precursor ions was used. In total, 35 LC-MS/MS runs were recorded consisting of 636,910 MS^1^ and 1,258,099 MS^2^ scans.

##### Data Analysis

LC-MS/MS files in RAW format were converted to mzML (18, 19) using Proteome Discoverer (version 1.4.0.0). Subsequently, pymzML (20) was used to convert the mzML files to the mascot generic format (mgf), if required. It is noteworthy that although MS1 scans were recorded in profile mode, all spectra used for quantification in pyQms need to be transformed to a centroided format, which can be done automatically during conversion to mzML or alternatively if mzML parsing is performed using pymzML (20).

All peptide identification algorithms were executed using Ursgal (21), a Python framework for performing peptide identifications, statistical postprocessing, and data visualization using unified parameters. Briefly, peptides were identified using the algorithms OMSSA (version 2.19, (22)), X!Tandem (version 2013.09.01, (23)), MS-GF+ (version 9979, (24)), and MyriMatch (version 2.1.138 (25)). Default values were used for most of the search parameters. Precursor mass accuracy was set to 5 parts per million (ppm), fragmentation mass accuracy was set to 20 ppm. Trypsin was defined as protease. A shuffled-peptide-based target–decoy database (conserving trypsin cleavage sites) was generated as described previously (17) using Ursgal. The *Chlamydomonas reinhardtii* database from the Joint Genome Institute version 5.3.1/236 (26) with Augustus 11.6 (27), the chloroplastic (28) and mitochondrial proteome as well as a contaminant database (cRAP, (29)) were used for the generation of the target decoy database containing in total 19,537 (target) protein sequences (see supplemental material). Further search parameters were variable modifications: oxidation of methionine (+15.9949 Da) and acetylation of the N terminus (+42.0106 Da). No fixed modifications were defined. Two missed cleavage sites were permitted. Database searches were conducted for ^14^N and ^15^N labeling separately. The posterior error probability was determined for each peptide-spectrum match using Percolator (version 2.08, (30, 31)). All peptide-spectrum matches with posterior error probabilities ≤1% at the level of the database search engine were used for all subsequent analyses. A total of 19,976 unique peptides were identified, mapping to 18,285 unique chemical formulas. The MS proteomics data have been deposited to the ProteomeXchange (32) via the PRIDE partner repository with the dataset identifier PXD003236. Venn diagrams of identified peptides and overlap of the different samples can be found in Supplemental Figs. 2 and 3, respectively. Spectrum annotations of proteins with one peptide-spectrum match or one distinct peptide can be found in the supplemental material. All identified proteins and peptides, including sequence coverage can be found in Supplemental Tables S2 and S3.

Peptides were quantified using pyQms (v0.5.0). Retention-time (RT) alignment and enhancement defining RT windows for all peptides (Supplemental Table S4) was carried out using piqDB as described earlier (17).

##### pyQms scoring

The pyQms matching score (mScore) is based on the work of Gower (33). The matching and scoring is performed on the *m/z* values and the intensity values independently yielding two scores, *i.e. S^mz^* and *S^intensity^*. In both cases, each peak *k* is scored, comparing the measured value *i* with the calculated value *j* (Equation 1), whereas a perfect match is 1. Each peak of the isotopologue that has a relative intensity (relative to the maximum intensity isotope peak) *r_k_* above the matching threshold (by default 1% of the maximum intensity isotope peak) is matched and scored.

(1) $$S_{ijk}\;\in \;\left[0,1\right]$$

##### The m/z *Score: S^mz^*

For each peak *k*, the *m/z* similarity between measured value *i* and the calculated value *j* is defined as

(2) $$S_{ijk}^{mz}=1-\left(\frac{\delta_{ijk}^{mz}}{\alpha }\right)$$

Whereas δ*_ijk_^mz^* the difference in ppm between measured *m/z_ik_* and calculated *m/z_jk_* and α defines the range in ppm, in which the score decreases from 1 to 0 in a linear fashion. In principle, α is equal to the precision of the measurement defined by the user (pyQms parameter REL_MZ_RANGE, default 5 ppm, http://pyqms.readthedocs.io/en/latest/params.html). For example, if the difference between measured and theoretical *m/z* values would be 2.5 ppm, then the *s_ijk_^mz^* score for this peak *k* would be 0.5.

The total *m/z* score for all peaks termed *S^mz^* is the weighted sum of all single similarity m/z scores *s_ijk_^mz^* (Equation 3). The weighting is defined by the theoretical intensity of the peak *k* relative to the highest peak in the theoretical isotope pattern, termed *r_k_*.

(3) $$S^{mz}=\frac{\sum_{}^{k}S_{ijk}^{mz}r_{k}}{\sum_{}^{k}r_{k}}$$

##### The Intensity Score: S*^intensity^*

Prior to intensity scoring, the scaling factor σ is calculated by comparing the intensities of the measured *i* and calculated *j* intensities for all peaks *k* within the matching threshold (see above). This scaling factor is calculated by dividing the weighted sum of the measured intensity by the weighted sum of the theoretical intensities (Equation 4).

(4) $$\sigma =\frac{\sum_{}^{k}intensity_{ik}r_{k}}{\sum_{}^{k}intensity_{jk}r_{k}}$$

Using this scaling factor, which is equal to the abundance of the measured molecule, one can calculate δ*_ijk_^intensity^*, which is the relative intensity error between measured and theoretical intensity for each peak k (Equation 5).

(5) $$\delta_{ijk}^{intensity}=\frac{\left|intensity_{ik}-\sigma \times intensity_{jk}\right|}{\alpha \times intensity_{jk}}$$

The intensity score of peak *k* is then defined (Equation 6).

(6) $$S_{ijk}^{intensity}=1-\left(\frac{\delta_{ijk}^{intensity}}{1-r_{k}+\epsilon }\right)$$

In analogy to the *m/z* score (*s_ijk_^mz^*), the denominator defines the range in which the peak-based intensity score decreases from 1 to 0. However, in contrast to the *m/z* score, the intensity error has to be weighted by the abundance of each peak (1 – *r_k_*) as more abundant peaks can be measured more accurately than smaller peaks. Additionally, we introduced ϵ (pyQms parameter REL_I_RANGE, default 0.2), which represents the most conservative relative error applied to the most precisely measured peak (*r_k_* = 1). Thus, the overall relative error (denominator) will increase with lower peaks (see online documentation http://pyqms.readthedocs.io/en/latest).

The total intensity score *S^intensity^* is the weighted sum of all similarity scores *k* in analogy to the *S^mz^* score:

(7) $$S^{intensity}=\frac{\sum_{}^{k}S_{ijk}^{intensity}r_{k}}{\sum_{}^{k}r_{k}}$$

##### The Combined Final Score: mScore

The final score is termed mScore and is a sum of *S^mz^* and *S^intensity^*. However, because some machines can measure *m/z* much more accurately then intensities, we introduced ξ to allow for flexibilities depending on the type of mass spectrometer used. ξ (the pyQms parameter MZ_SCORE_PERCENTILE, default 0.4) is the fraction the *S^mz^* score is weighted into the sum. Thus, the final mScore is defined as

(8) $$mScore=\xi S^{mz}+\left(1-\xi \right)S^{intensity}$$

##### Statistical Evaluation of the Gold Standard Data Set

Statistical evaluation was performed by reducing and grouping the matched isotope pattern chromatograms (MICs). A MIC is defined as all spectra matches of a peptide with a certain charge state in one LC-MS/MS run. As the name suggests, an MIC is similar to a XIC but based on all matched isotope patterns instead of a single *m/z* value, thus containing an additional data dimension, that is, the mScore. Grouping of the MICs was based on the chemical formula and charge of the respective peptide. First, each MIC was reduced to one single match, which showed the highest mScore, yielding two values per MIC (*i.e.* score and intensity). Second, the reduced MICs belonging to one chemical formula and charge state were grouped depending on their ^15^N labeling into one of 11 label percentile bins ranging from 0–5, 6–15, 16–25, …, 96–100%. Third, each bin was reduced to a single match based on the maximum score. Finally, for each mixed sample, we considered only one ground truth at a time. Given this setup, we reduced the evaluation of a quantified chemical formula and charge combination for each ground truth in each sample to ten bins, for which the true and false positives/negatives were counted at different score thresholds. For this, MICs (with a certain charge in one MS run) were grouped as following: for true positives (TP), the expected labeling percentile bin has matches, and no other bin has matches; for false positives (FP), the expected bin has no matches, but another unexpected bin has matches; for false negative (FN) the expected bin has no match at all, and for true negative (TN) the unexpected bin has no match at all. False discovery rate (FDR) was defined as FP/(FP+TP), true positive rate (TPR, sensitivity) as TP/(TP+FN) and false positive rate (FPR) as FP/(FP+TN). In total, 13.9e^6^ MICs were grouped according to molecular formula, charge state, and LC-MS/MS run.

##### Label-Free Quantification

The data set from Bruderer *et al.* (34) was used to evaluate label-free peptide quantification performance and quality of pyQms for data-dependent acquisition (DDA)^1^ and data-independent acquisition (DIA) mode. RAW MS files were obtained from www.peptideatlas.org and converted into mzML using msconvert, which is part of Proteowizard (version 3.0.7408, (35)). MaxQuant/Andromeda (version 1.4.1.2, (5)) peptide identification results were used from Bruderer *et al.* (34) and stored in piqDB. All peptides were subsequently quantified by pyQms using default parameters except an adjustment of the machine offset in ppm to correct for measuring error (21, 34). Carbamidomethylation of cysteine was defined as fixed modification. RT alignment (Supplemental Table S5), and enhancements were carried out using piqDB as described earlier (17, 36). Intensity alignment (Supplemental Table S6) of all samples was performed as described earlier (37). The signal intensity of a given peptide (*i.e.* peptide charge combination) within each MS run was defined as the maximum intensity over all spectra within the predefined retention time window. RT alignment functions and raw peptide amounts for the DDA data set can be found in Supplemental Tables S5 and S7, respectively. The linear correlation of peptide concentrations and their matched peptide intensities over a wide concentration range (0.8 fmol/μl–819.2 fmol/μl) can be found in Supplemental Figs. 4 and 5.

Peptide ratios (log_2_) between LC-MS/MS runs were calculated using these raw quantification data (mScore > = 0.8). MaxQuant raw peptide quantification results were obtained from the supplement material of Bruderer *et al.* (34). Correlation plots between the MaxQuant and pyQms amounts for the spiked-in proteins and for the human background proteins can be found in Supplemental Figs. 6 and 7, respectively. In order to compare pyQms with MaxQuant, the peptide (peptide charge combinations) ratios were equally calculated using the raw peptide amounts reported by Bruderer *et al.* (34).

A similar procedure was applied to the DIA measurements of the same samples. For all peptides that are proteotypic to the spiked-in proteins, all fragment ions were determined and ions suitable for quantification were determined using an algorithm that will be described elsewhere. These selected fragment ions were quantified in the cycle window corresponding to their peptide precursor *m/z*. A complete table of all ions used for quantification can be found in Supplemental Table S8. DIA runs were intensity aligned with the same method as for the DDA runs as described earlier (37). DIA peptide quantification was based on summing up all fragment ion intensities (maximum intensity in each MS run within the retention time window). Only ions that could be quantified in both samples were taken into account for the ratio calculation. All pyQms peptide quantification results for the DIA data set can be found in Supplemental Table S9. Spectronaut DIA raw peptide amounts were taken from the supplement material of Bruderer *et al.* (34), and those were equally summed up at the peptide level. Finally, to ensure comparability, peptide ratios (log2) were calculated similar as for the pyQms results.

##### Peptide Ratio Evaluation

The data set published by Bruderer *et al.* (34) contains 12 non-human proteins spiked in a background of human HEK293 cells. The setup contained three different master mixes (MMs) spanning several orders of magnitudes of various protein concentration (MM1: 1.1 to 13.33 fold, MM2: 1- to 200-fold, MM3: 1- to 16,384-fold; eight concentrations in total for each MM). Three technical replicates for each of the eight different samples containing a distinct concentration of each master mix were measured by LC-MS/MS (Supplemental Fig. 1). pyQms was used to quantify the peptides belonging to the spiked-in proteins. Subsequently, the quantification was used to calculate ratios for all peptide charge combinations between all samples (see above), including all technical replicates. This resulted in 276 potential ratios for each peptide (defined by all combinations of the 24 measurements). All peptides belonging to one spiked-in protein were used to evaluate the observed and calculated log_2_ ratio against the known ground-truth using a two-sided *t* test implemented in Scipy (www.scipy.org, (38)). At least three peptides (or peptide charge combination ratios) were required per sample comparison. We grouped the calculated *p* values into four bins that classify the quality of the quantification, *i.e.* the similarity to the expected ground truth: a *p* value (1) above 0.05 was classified as 'not significant different' and colored blue, (2) 0.05–0.01 (*) colored green, (3) 0.01–0.001 (**) colored yellow, and (4) smaller than 0.001 (***) colored red. This color scheme is consistent throughout this work (Fig. 2 and Supplemental Figs. 8–10).

##### Liquid Chromatography MS of Ribonucleosides

Chemically synthesized MS-grade adenosine (C_10_O_4_N_5_H_13_; Carbosynth, Ltd., Berkshire, UK) was dissolved in 5 mm ammonium formate, pH 5.3, at a concentration of 50 ng/μL and further diluted to yield the following concentrations; 1000 pg/μL, 500 pg/μL, 250 pg/μL, 125 pg/μL, 62.5 pg/μL, 31.25 pg/μL, 20 pg/μL, 10 pg/μL, 5 pg/μL, 2.5 pg/μL, 1 pg/μL, 0.5 pg/μL, 0.2 pg/μL, 0.1 pg/μL, 0.05 pg/μL, 0.02 pg/μL, and 0.01 pg/μL. 4 μL of each dilution was subjected to reversed phase LC-MS analysis on a self-packed 75 μm × 500 mm porous graphitic carbon column connected to a Q Exactive mass spectrometer (Thermo Scientific). Full MS spectra (*m/z* 100–700) were recorded with three technical replicates for each sample, and the resulting LC-MS runs were analyzed by pyQms. Full details of the chromatography and MS conditions will be published elsewhere.

##### Experimental Design and Statistical Rationale

The pulse (-chase) gold standard data set contains in total six samples (0, 20, 40, 60, 80, and 100% ^15^N labeling) representing six biological replicates. These samples were mixed (0I100, 20I100, 40I100, 0I60, and 0I80), resulting in three technical replicates for the 0% sample and in two technical replicates for the 100% sample. All other samples have no technical replicate within these mixes. Furthermore, each of the seven filter-aided sample preparation fractions (16) was measured individually in order to achieve higher sensitivity. In total 35 LC-MS/MS runs, including 636,910 MS^1^ and 1,258,099 MS^2^ scans of the pulse gold standard data set, were evaluated. Please refer to the methods section “Statistical Evaluation of the Gold Standard Data Set” for all statistical tests used for the analysis of this data set.

##### Requirements, Availability, and Documentation

pyQms requires Python 3.4+ and is platform independent (OS X, macOS, Linux, and Windows). The module is freely available on https://github.com/pyQms/pyqms or pypi, published under Massachusetts Institute of Technology (MIT) license and requires no additional modules to be installed. We recommend pymzML (20) for fast access to spectra from mzML files. To run example scripts, it is necessary to install pymzML or to change the code for alternative spectrum access. Some scripts also require the openpyxl or rpy2 modules.

The documentation of pyQms including parameter description (http://pyqms.readthedocs.io/en/latest/params.html), a quick-start tutorial (http://pyqms.readthedocs.io/en/latest/quick_start.html), and example scripts (http://pyqms.readthedocs.io/en/latest/example_scripts.html) can be found online. pyQms can be run on standard desktop computers. For further hardware requirements, please refer to the online documentation.

## RESULTS

### 

#### 

##### Algorithm Design

**Fig. 1.**
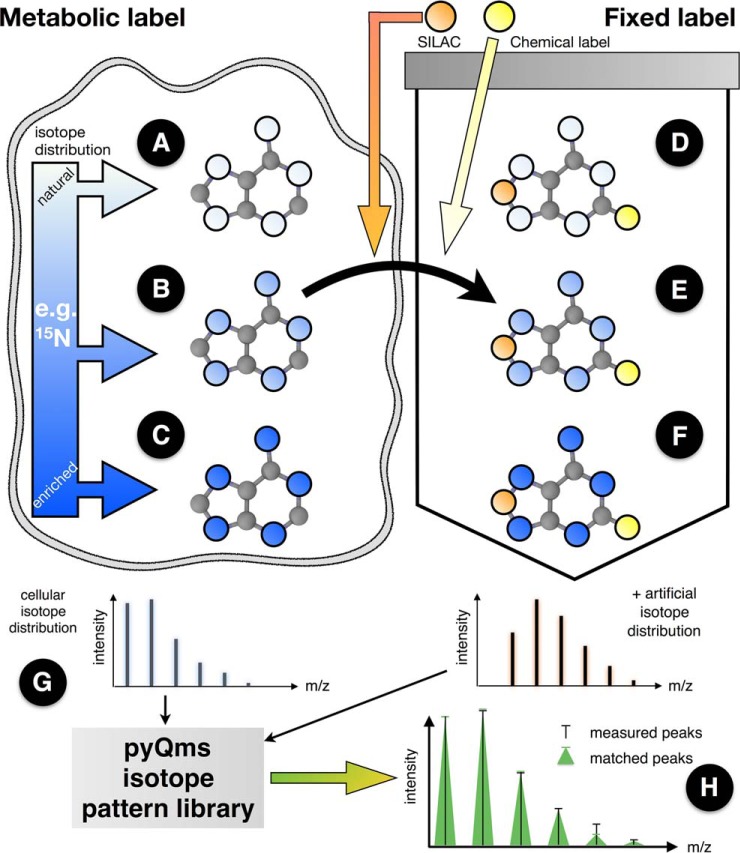
Labeling strategies employed in mass spectrometry separated into metabolic (*left*) and fixed labels (*right*). Metabolic labeling (*e.g.* ^15^N salt or ^13^C sugar) is metabolized in the cell and incorporated into newly synthesized molecules. The isotope distribution of the labeled element can be natural (*A*, white circles), partially enriched by an isotope (*e.g.* ^15^N, light blue color represents *e.g.* an average labeling of 60%, *i.e.* three of five nitrogens are ^15^N) as observed during pulse or pulse-chase experiments (*B*, light blue) or fully enriched (*C*, dark blue). Fixed labels are incorporated into or attached to the molecule during or after the synthesis steps. Fixed labeling can be performed *in vivo* (*e.g.* stable isotope labeling with amino acids in cell culture incorporation (2)) or *in vitro* (*e.g.* digestion in ^18^O-labeled water (45)). In both cases the element isotope distributions of the label are independent of the cellular distributions and are thus treated as different element pools (*D–F*). Combining different labeling strategies permits novel multiplexing strategies. Only pyQms can be used to quantify all six cases (*A–F*) in all variations and combinations irrespective of the label or the molecule type and to, most importantly, score the quantifications. The metabolic isotopic distribution (left isotopologue) and the artificial isotopic distribution (right isotopologue of a potential fixed label is used to calculate an accurate isotope pattern for each molecule (*G*). These patterns are compared with the MS measurements (*H*). Matches are evaluated providing the similarity match score (mScore). Black bars, measured peaks; green triangles, matched peaks; *x*-axes, *m/z* value; *y*-axes, intensity. For considerations on the terms fixed and metabolic labeling, please refer to the online methods.

The core of pyQms is a standalone open source Python module that can be incorporated easily into any analytical workflow, can be run on standard desktop computers, and that benefits from the resources of a rich scientific computing community (20, 21, 39–42). The workflow of pyQms can be divided into two steps. First, an isotope pattern library is built, based on user-defined molecules (Fig. 1*G*). These molecules are specified as chemical formulas or peptide sequences, optionally with modifications following the unimod standard. The metabolic isotopic distribution and the artificial isotopic distribution of the fixed label are taken into account. pyQms uses this information to calculate an accurate isotope pattern for each molecule based on its elemental composition, the isotope distributions, and the respective masses. Second, the predicted isotope patterns are compared with the MS measurements (Fig. 1*H*) and scored by calculating a similarity coefficient (33) optimized for MS data yielding a similarity match score, termed mScore. The matching and scoring algorithm initially uses the *m/z* values and the intensity values independently and combines both later, offering the possibility to adjust the scoring algorithm to the machine type, as some instruments can measure *m/z* values much more accurately than intensities. Furthermore, the similarity between each measured and calculated isotope pattern peak is weighted by its relative abundance. Thus, the more abundant peaks contribute more to the mScore (see M&M for details). We developed this similarity matching approach as the basis for pyQms since it provides several advantages: (i) The isotope pattern contains multiple peaks that are all used for quantification, thus increasing the robustness over using only a single value. (ii) The matching approach tags each quantification with a quality assessment reflected by its mScore. (iii) Coeluting molecules can either be distinguished reliably since the isotope patterns differ or they receive a low mScore. (iv) The matching algorithm can adapt to any mass spectrometer accuracy. Thus, future technological advancement in machine accuracy will automatically translate into higher matching sensitivity without the need to adjust the algorithm. Overall, any MS data analysis workflow that relies on single *m/z* observations will benefit from the incorporation of isotope pattern matching and our scoring methodology.

Furthermore, pyQms accepts so called evidence files as input (*e.g.* peptide identifications from Ursgal (21) or manually curated data). These files allow molecules with the identical chemical formula but different identities to be distinguished by associating their identity with a given retention-time window. pyQms offers a modular system to use custom functions that can be used to determine the abundance of a given molecule. The basic built-in function determines abundance by the maximum intensity within a retention-time window that is defined in the evidence files. However, pyQms was primarily build for bioinformaticians, thus functions to define retention windows or to determine abundances can easily be incorporated if required.

##### pyQms Provides Quantification with High Accuracy and Sensitivity

**Fig. 2.**
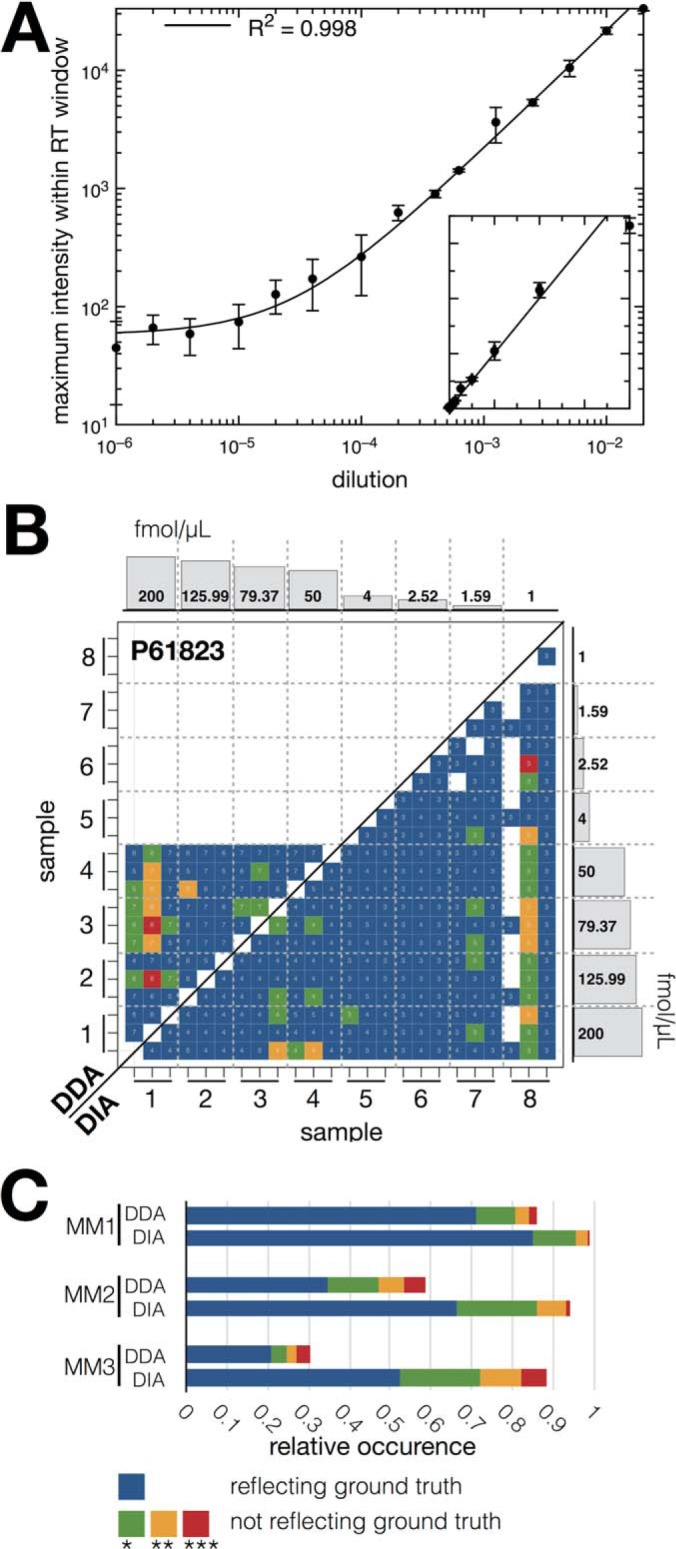
**Label-free pyQms quantification of metabolites and peptides.** (*A*) Dilution series of chemically synthesized adenosine (axes plotted in log scale), each dilution comprising three technical replicates. Line shows the linear regression function (R^2^ = 0.998). *Inset* shows axes plotted in linear scale; *x*-axes, dilution of nucleoside; *y*-axes, maximum intensity. (*B*) Example of the statistical evaluation of pyQms peptide quantifications of the Bruderer *et al.* data sets (DDA, *top left*; DIA, *bottom right*). Shown is the heat map for spiked-in protein P61823 (ribonuclease pancreatic, *Bos Taurus*, master mix 2). The colors reflect the *p* value for the two-sided *t* test obtained after testing whether the calculated peptide ratios between two samples (*x* and *y* axes, eight samples, ticks represent each three technical replicates) differ significantly from the ground truth ratios. Histograms at the axes show the spiked-in protein concentrations for the eight samples (master mix 2, for sample setup and other master mix composition refer to Supplemental Fig. 1 and Bruderer *et al.* (34), *x*-axes, sample; *y*-axes, concentration of spiked-in proteins. The number of peptide charge combination ratios (for DDA samples) or peptide ratios (DIA) used for the *t* test are shown as numbers in the bins. (*C*) Stacked bar plot for relative occurrences of the *p* values over all 12 spiked-in proteins across all three master mixes (MM1, MM2, and MM3). Legend: *x* axis, relative occurrence of *p* value; *y* axis, master mix, and data acquisition method combination (DDA or DIA). *p* value legend for (*B*) and (*C*), blue: reflecting the ground truth; green (*p* value ≤0.05), yellow (*p* value ≤0.01), and red (*p* value ≤0.001): not reflecting the ground truth.

To assess the accuracy and sensitivity of pyQms for metabolomics, we analyzed chemically synthesized MS-grade adenosine in a dilution series ranging from 0.04–4000 pg/μL (Fig. 2*A*). Importantly, the quantification remained linear over a broad range of concentrations (four to five orders of magnitude) with an R^2^ of 0.998, showing that pyQms can be used for the sensitive quantification of metabolites.

Next, to compare pyQms against established quantification tools in proteomics, we used the data sets of Bruderer *et al.* (34) that contain eight biological replicates of human HEK293 cells, each measured three times in DDA and DIA mode. We compared the published quantification results of the DDA data set analyzed with MaxQuant (5) and of the DIA data set analyzed with Spectronaut (43) to our results obtained with pyQms (Fig. 2*B*).

We found that 17.4% of the comparisons reflect the ground truth in the DDA samples. However, concentrations below 4 fmol/μL could not be quantified confidently, which is similar to the results reported for MaxQuant (5) (21.3%, Supplemental Fig. 8, 9I2). Furthermore, we found that 82.2% of the ratios obtained with pyQms reflect the ground truth for the DIA data set (Fig. 2*B*, *bottom right half*), similar to what was reported for Spectronaut (43) (76.8%, Supplemental Fig. 8, 9I3). These results are comparable to the remaining spiked-in proteins (Fig. 2*C*). In summary, pyQms quantifications reflected 71.2%/85.2% (*n* = 1380 ratios) of the ground truth in the DDA/DIA runs for master mix 1 (MM1), 34.9%/66.3% (*n* = 1380 ratios) for MM2, and 20.8%/52.5% (*n* = 552 ratios) for MM3 (Fig. 2*C*), which is similar to the results reported for MaxQuant and Spectronaut (34) (MM1: 62.8%/78.8%, MM2: 23.1%/68.5%, MM3: 15%/72.1%) (Supplemental Figs. 8–11, Supplemental Tables S10 and S11). This shows that pyQms covers the detection limits of data acquired in DDA mode or DIA modes.

##### Quantification of Pulse (-Chase) Samples

**Fig. 3.**
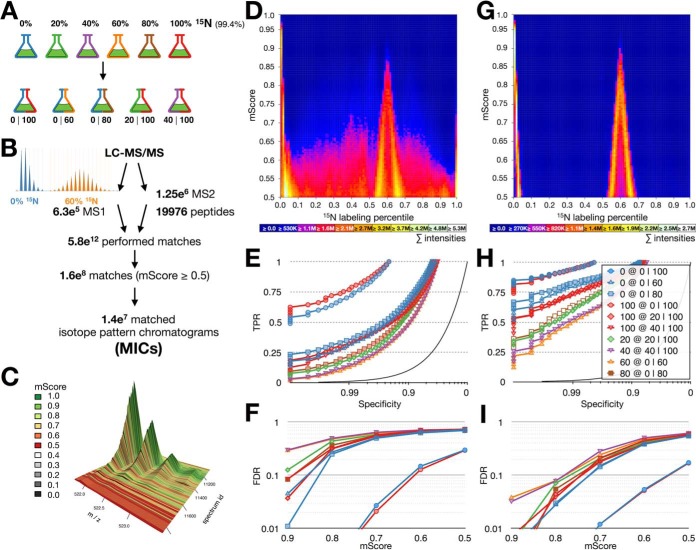
**The partially labeled proteome gold standard.** (*A*) Cultures of *C. reinhardtii* were grown on medium containing 20, 40, 60, 80, and 100% ^15^N. Each partially labeled proteome was mixed with an unlabeled (^14^N) or fully labeled (^15^N) proteome, yielding five mixed samples (0 100, 0 60, 0 80, 20 100, and 40 100). (*B*) Unbiased quantification workflow. All identified peptides (19,976 peptides, 18,285 distinct chemical formulas) were quantified in all MS^1^ spectra (6.3e^5^) of all LC-MS/MS runs in five charge states performing in total 5.8e^12^ matches. Matches were filtered using an mScore threshold of 0.5 and assembled into 1.4e^7^ MICs. Example isotopologues for 0 and 60% ^15^N incorporation are shown on the *left*. (*C*) 3D visualization of a typical MIC, colors indicate mScores for each match within the MIC; *x* axis, *m/z*; *y* axis, spectrum id; *z* axis, intensity. (*D*) Visual evaluation of all matches in the 0 60 sample, *x* axis labeling percentile, *y* axis mScore, heat equals summed up intensities of matches in all MS^1^ spectra in a given bin (representing all identified and quantified peptides in all matched charge states). (*E*) ROC curves of pyQms performance in all samples shown as specificity (*x* axis, log scale) versus true positive rate (*y* axis). (*F*) mScore-dependent FDR; *x* axis, mScore; *y* axis, FDR (log scale). (*G-I*) as (*D-F*), but quantifications are limited to retention time windows. Legends (*D*, *E*, *G*, *H*): 0% (blue), 100% (red), 20% (green), 40% (purple), 60% (orange), 80% (brown); mixtures: 0I100 (circles), 0I60 (triangles), 20I100 (diamonds), 0I80 (squares), 40I100 (reverse triangles).

In order to evaluate the quantification performance of pyQms for pulse (-chase) samples, we create a gold-standard data set of well-defined partially labeled proteomes. This data set can be used to benchmark the accuracy of any quantitative software aiming at analyzing partially labeled proteomes. We cultivated the green algae *Chlamydomonas reinhardtii* in media containing 0 to 100% ^15^N metabolic label in 20% increments. Extracted proteins were combined to create five mixes: 0I100, 20I100, 40I100, 0I60, and 0I80 (Fig. 3*A*), which were analyzed by LC-MS/MS. Subsequently, we quantified all identified peptides, allowing all possible combinations of charge states and all ^15^N enrichment percentiles to be matched in all MS^1^ scans. This summed up to a total of 5.8e^12^ performed matches, which resulted in 1.6e^8^ positive matches with mScores ≥0.5. We grouped matches with an identical chemical formula, charge state, and labeling percentile from one LC-MS/MS run into MICs. Altogether, we found 1.4e^7^ MICs. For each sample, we assessed the matching quality by aggregating the single matches of all MICs into one heat map (Fig. 3*D*, Supplemental Fig. 12). We used the ^15^N-labeling percentile and the mScore of each match as coordinates for the heat map bin and added the abundance of that match to this bin (including peptide abundances of all spectra assembled in a MIC). While the 0I100 mix can be separated reliably (Supplemental Fig. 12*A*), this was not the case for the other mixes (Supplemental Figs. 12*C*, 12*E*, and 12*G*). Even though it was possible to identify the ground truth, the correct result was obscured by false positives leading to a high background signal that was distributed over all labeling percentiles.

We statistically evaluated our results by creating receiver-operating characteristics (ROC) of the MICs against the ground truth for each mixed sample (Fig. 3*E*). The ROC curves show that pyQms reliably matches the 0I100 mix (71.1% and 76.9% TPR at 99% specificity, blue and red circles). In contrast, the partially labeled samples result in TPRs between 12.7% and 21.5% at 99% specificity. The fully labeled (100%) or unlabeled samples (0%), if mixed with a partially labeled sample, have lower ROC curves compared with their counterpart in the 0I100 mix (30.9%–35.4% TPR at 99% specificity). The mixes containing partially labeled samples show a high FDR even at very high mScores (*e.g.* the 0 and 60% samples show FDRs of 4.5% and 29.3%, respectively, at mScores ≥ 0.9 in the 0I60 mix; Fig. 3*F*). Only in the 0I100 mix, we detected FDRs of 2.7% and 2.1% for the 0 and 100% samples, respectively, at mScores ≥ 0.7.

##### Decreasing the FDR in Pulse (-Chase) Experiments

To obtain a lower FDR, we limited quantification to stringent (2–2.5 min) RT windows (Supplemental Fig. 13, Supplemental Table S4) based on the RT alignment strategy described earlier (17). This strategy significantly reduced FP matches, as shown by reduced noise in the heat map (Fig. 3*G*). Furthermore, the ROC curves improve significantly for all five mixes (Fig. 3*H*). Finally, the mScore-dependent FDR (Fig. 3*I*) shows rates of 3.2% and 3.8% for the 40% (40 100 mix) and 60% (0 60 mix) samples, respectively, at very stringent mScores of ≥0.9 (Fig. 3*I*). This shows, that applying RT windows can significantly reduce the FDR in pulse (-chase) experiments.

## DISCUSSION

### 

#### 

##### pyQms Performs State-of-the-Art DDA and DIA Quantification

The presented results emphasize that pyQms performs optimally within the detection limits of data acquired in DDA mode or DIA modes similar to what is regarded as state-of-the art in proteomics. However, pyQms combines the functionality to quantify both DDA and DIA data in a single software. Furthermore, pyQms uniquely offers a highly accessible bioinformatics library, so other packages can incorporate its isotope matching procedure to increase their matching quality with ease. Ultimately, isotope pattern matching will replace approaches relying on single *m/z* values, especially in the advent of the broad availability of high-resolution mass spectrometers.

##### Benchmarked Partial Label Quantification

To demonstrate that pyQms goes beyond the current state-of-the-art, we analyzed a data set of differentially labeled proteomes, like observed during pulse or pulse-chase experiments. This reflects the ultimate challenge for any quantification algorithm since each molecule has different enriched isotope incorporation levels depending on the time of synthesis. Thus, one single labeling state cannot be observed, complicating analyses for several reasons: First, the isotope distribution is different for each labeling state, yet, it is difficult to distinguish closely related labeling states. Each isotope pattern must therefore be evaluated independently or in a mixed model. Second, partially labeled molecules broaden the isotope pattern and lead to more peaks, which are consequently less intense (Supplemental Fig. 14). This effect leads to a loss of signal and quantification accuracy. Finally, the number of isotope patterns that need to be matched increases by two orders of magnitude when compared with label-free quantification, thus requiring significant computational resources.

Our pulse or partially labeled gold standard data set revealed a low TPR for samples containing partially labeled peptides at high specificities in contrast to a sample containing only unlabeled or fully labeled proteins (Fig. 3*E*). This confirms that the complexity of the sample and the number of detected peaks strongly influences the classification. Similarly, this is reflected in the mScore-dependent FDR (Fig. 3*F*). These results illustrate the challenge of assessing partially labeled molecules in general. These difficulties are rarely addressed as they only become apparent by benchmarking against a defined partially labeled sample. Our gold standard data set can therefore be used to benchmark new software tools for the quantification of pulse and pulse-chase data.

The benchmarking results of pyQms against the gold standard data set underlines the universal application and high quality of pyQms, which goes beyond what is currently available in single algorithms.

##### pyQms Is Suitable for Large-Scale Pulse (-Chase) Studies

The approach to lower the FDR and increase the TPR by applying stringent RT windows demonstrates that pyQms allows accurate large-scale high-throughput quantification of pulse (-chase) MS experiments to be performed. Interestingly, the sample resembling the most common proteomic samples (0I100 mix) shows an FDR of only 1.2% at mScores ≥ 0.7 using accurate isotope pattern matching in combination with RT windows (Fig. 3*I*, blue and red circles). This indicates that pyQms could be sufficient for MS^1^-based identifications and quantification in proteomics. Importantly, this should also pave the way to reliably overcome the undersampling issue in proteomics (44), especially if one takes the mScore as a quality criterion into account. In fact, if the Bruderer *et al.* (34) data set is evaluated with respect to mScore, this effect becomes very obvious (Supplemental Fig. 15).

## CONCLUSIONS

In conclusion, we have demonstrated that pyQms is a powerful software to quantify any molecule measured by MS, independent of the type of molecule, label, research field, or acquisition mode. These unique features enable novel experimental designs and multiplexing strategies, which are urgently required in the era of personalized medicine in order to simultaneously compare hundreds of clinical samples. Using pyQms, researchers will be able to quantify MS data from proteomics, metabolomics, lipidomics, glycomics, or other research fields with a single, universal software solution. pyQms is open source and freely available via https://github.com/pyQms/pyqms under an MIT license.

## Data availability

The mass spectrometry proteomics data have been deposited to the ProteomeXchange (32) via the PRIDE partner repository (http://www.ebi.ac.uk/pride/archive/) with the data set identifier PXD003236.

## Supplementary Material

Supplemental Data

## Footnotes

^1^ The abbreviations used are:

DDA: data-dependent acquisition
DIA: data-independent acquisition
FDR: false discovery rate
FN: false negative
FP: false positive
FPR: false positive rate
LC-MS/MS: liquid chromatography coupled tandem mass spectrometry
MIC: matched isotope pattern chromatogram
MS: mass spectrometry
ROC: receiver-operating characteristic
RT: retention time
TAP: tris-acetate-phosphate
TN: true negative
TP: true positive
TPR: true positive rate.
