# Supplementary material for: pyQms enables universal and accurate quantification of mass spectrometry data
Source: Mol Cell Proteomics. 2017 Jul 20;16(10):1736–45. doi: 10.1074/mcp.M117.068007 (PMC5629261; doi:10.1074/mcp.M117.068007)
Supplement: Supplemental Data [file supp_16_10_1736__index.html]

pyQms enables universal and accurate quantification of mass spectrometry data — pyQms enables universal and accurate quantification of mass spectrometry data — Universal quantification tool for mass spectrometry — Supplemental Data 

# pyQms enables universal and accurate quantification of mass spectrometry data

## Supplemental Data

- Supplemental Data (.zip, 50.8 MB) - Annotated peptide spectra as required by MCP guidelines.
- Supplemental figures (.pdf, 6.6 MB) - Supplemental figures 1 - 15 including descriptions.
- Supplemental methods (.pdf, 83 KB) - Supplemental methods.
- Supplemental table descriptions (.pdf, 66 KB) - Supplemental table descriptions for STable 1 - 12
- Supplemental Table S1 (.xlsx, 20 KB) - Overview of quantification engines for DDA and DIA mass spectrometry data.
- Supplemental Table S2 (.xlsx, 1.9 MB) - All identified peptides in the pulse gold standard data set.
- Supplemental Table S3 (.xlsx, 365 KB) - Identified peptides and protein sequence coverage.
- Supplemental Table S4 (.xlsx, 2.7 MB) - Retention time windows.
- Supplemental Table S5 (.xlsx, 455 KB) - Retention time alignment functions for the Bruderer et al. dataset.
- Supplemental Table S6 (.xlsx, 10 KB) - Intensity alignment correction factors.
- Supplemental Table S7 (.xlsx, 89 KB) - DDA quantification results for the spiked-in-proteins of the DDA data set from Bruderer et al. (2015).
- Supplemental Table S8 (.xlsx, 738 KB) - Complete curated spectral library used for the DIA quantification approach.
- Supplemental Table S9 (.xlsx, 56 KB) - DIA quantification results for the spiked-in-proteins of the DIA data set from Bruderer et al. (2015).
- Supplemental Table S10 (.xlsx, 9 KB) - T-test results on master mix level for the DDA and DIA approaches by pyQms.
- Supplemental Table S11 (.xlsx, 11 KB) - T-test results on protein level for the DDA and DIA approaches by pyQms.
- Supplemental Table S12 (.xlsx, 10 KB) - Header/sample descriptions for the supplemental table headers.
- Fasta database (.zip, 16.4 MB) - C reinhardtii fasta database for pulse chase gold standard dataset
