# Supplementary material for: pyQms enables universal and accurate quantification of mass spectrometry data
Source: Mol Cell Proteomics. 2017 Jul 20;16(10):1736–45. doi: 10.1074/mcp.M117.068007 (PMC5629261; doi:10.1074/mcp.M117.068007)
Supplement: Supplemental Data [file 10.1074_M117.068007_mcp.M117.068007-2.pdf]

Supplemental Figure 1

Data set overview

Data set and analysis overview used for the evaluation of pyQms. A-C; Summary of the Bruderer *et al.* (2015) data set evaluation. D-J; Summary of the pulse chase gold standard data set evaluation (Leufken *et al.* 2017).

A) Sample and master mix overview. Eight different samples were generated containing an equal background of human proteins from HEK293 cell lysates (0.5 μg/μl). Additionally, three master mixes of non-human proteins were diluted as indicated and spiked into the samples (resulting protein concentrations are indicated, y-axis: log scale). B) Data acquisition and analysis performed by Bruderer *et al.* (2015). Samples were measured using DDA or DIA measuring mode. Subsequently, data were analyzed with MaxQuant (DDA measurements only, Andromeda used for identification) and Spectronaut (DIA measurements only). pyQms was used for peptide quantification of both DDA and DIA measurements. C) Summary of the peptide ratio evaluation principle using pyQms as quantification engine.

D) The green algae *C. reinhardtii* was grown several generations in medium containing different proportions of ^{15}N (0, 20, 40, 60, 80, 100 %) and E) mixed on equal chlorophyll level. F) Full cell lysates were tryptically digested and subjected to LC-MS/MS analysis. G) LC-MS/MS files were analyzed using Ursgal, employing four different database search engines for peptide identification in unlabeled or fully labeled state (100% ^{15}N). Resulting peptides were quantified using pyQms and further analysed using the analysis framework piqDB. I) Example match for the 0l60 mixture sample displaying the two isotopologues. J) All matches (in each mixture peptide isotopologues from 0 - 100 % in 1 % steps were quantified) were used to determine the mScore dependent false discovery rate of pyQms.

Bars on the sides indicate which parts of the data acquisition and analysis was performed by Bruderer *et al.* (2015) or within this manuscript (Leufken *et al.* 2017).

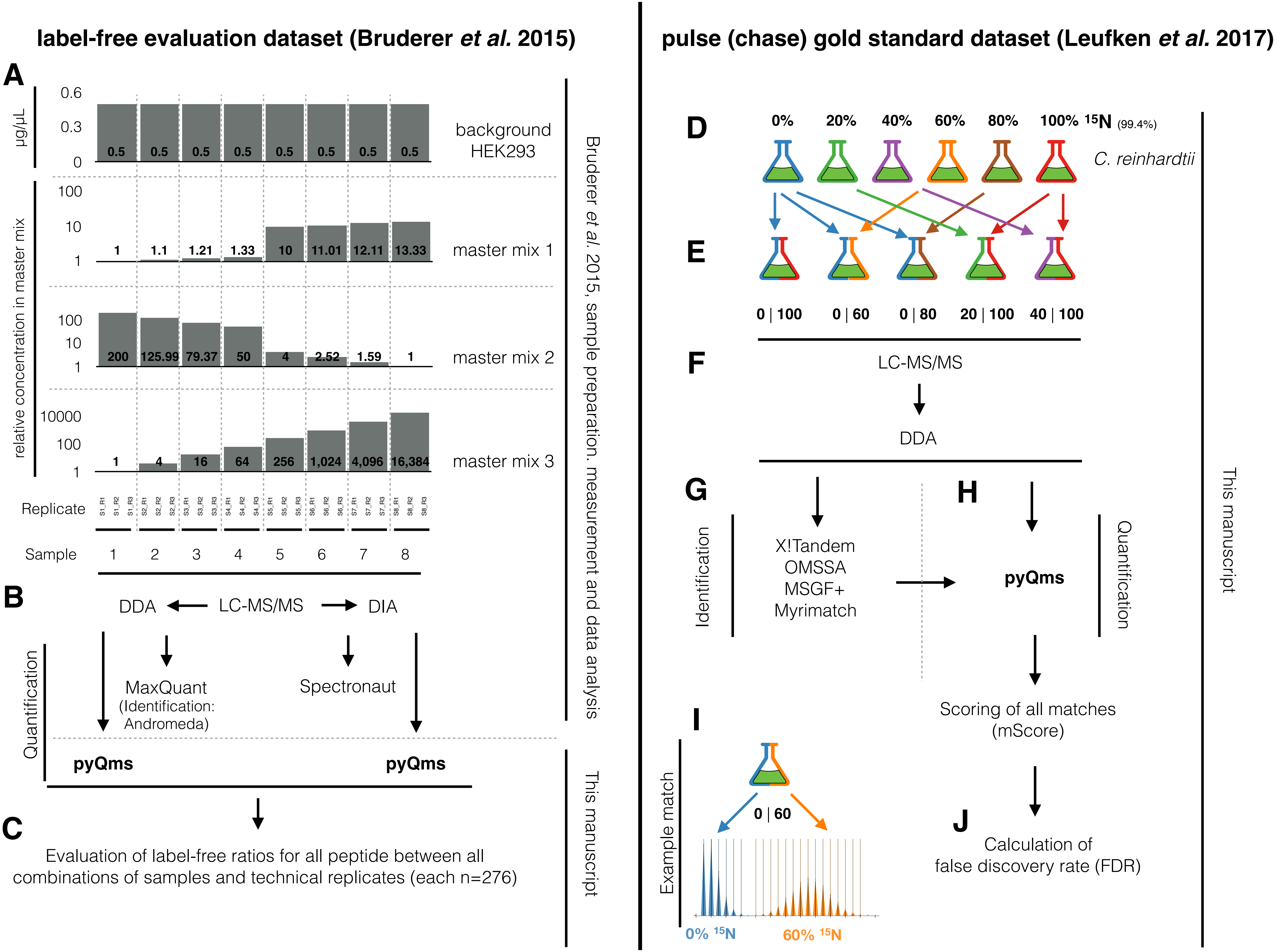
label-free evaluation dataset (Bruderer et al. 2015)
pulse (chase) gold standard dataset (Leufken et al. 2017)
A
µg/µL
background HEK293
relative concentration in master mix
master mix 1
1
1.1
1.21
1.33
10
11.01
12.11
13.33
master mix 2
200
125.99
79.37
50
4
2.52
1.59
1
master mix 3
1
4
16
64
256
1,024
4,096
16,384
Replicate
Sample
B
DDA
LC-MS/MS
DIA
Quantification
MaxQuant
(Identification: Andromeda)
Spectronaut
pyQms
pyQms
C
Evaluation of label-free ratios for all peptide between all combinations of samples and technical replicates (each n=276)
Bruderer et al. 2015, sample preparation, measurement and data analysis
This manuscript
D
0%
20%
40%
60%
80%
100% 15N (99.4%)
C. reinhardtii
E
0 | 100
0 | 60
0 | 80
20 | 100
40 | 100
F
LC-MS/MS
DDA
G
H
Identification
X!Tandem
OMSSA
MSGF+
Myrimatch
pyQms
Quantification
Scoring of all matches (mScore)
I
Example match
0 | 60
0% 15N
60% 15N
J
Calculation of false discovery rate (FDR)
This manuscript

Supplemental Figure 2

Identified peptides in partially labeled gold standard data set

Venn diagram of peptide overlap obtained with the four used peptide database identification engines (OMSSA, red; X! Tandem, yellow; Myrimatch, blue; MS-GF+, purple) in the Python framework Ursgal (Kremer *et al.* 2016) with a posterior error probability (PEP) <= 1% on database search engine level. ^{14}N and ^{15}N identifications were pooled for each engine. For details on the peptide identification please refer to the methods section.

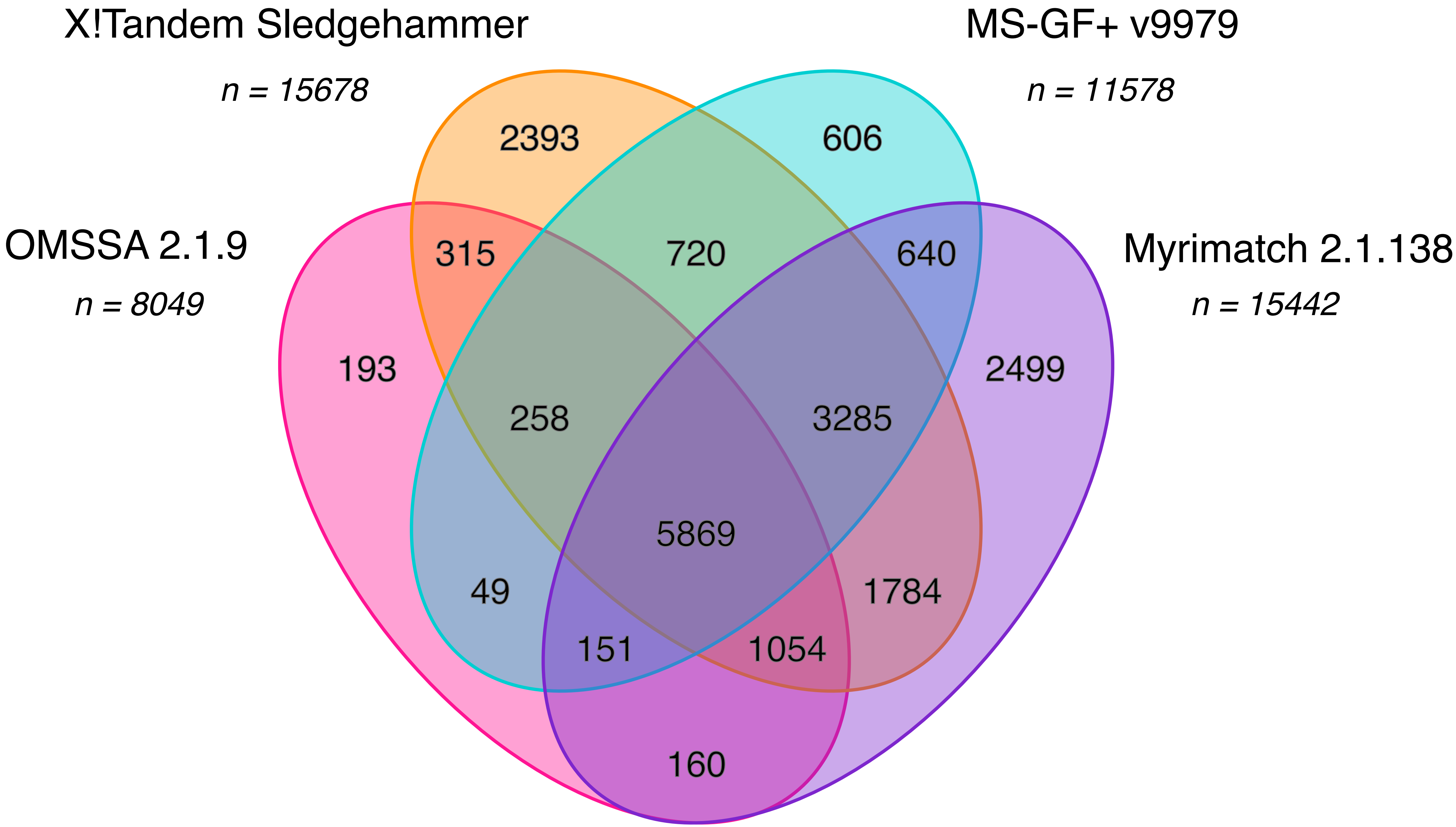
X!Tandem Sledgehammer
n = 15678
MS-GF+ v9979
n = 11578
OMSSA 2.1.9
n = 8049
Myrimatch 2.1.138
n = 15442
2393
606
315
720
640
193
2499
258
3285
5869
49
1784
151
1054
160

Supplemental Figure 3

Identified peptide overlap between partially labeled gold standard data set samples

Venn diagram of peptide overlap between the five partially labeled gold standard data sample sets. ^{14}N and ^{15}N identifications were pooled and the overlap determined using Ursgal (Kremer *et al.* 2016). A posterior error probability (PEP) <= 1% on database search engine level is assured. 0l100, mixture of ^{14}N and 100% ^{15}N samples; 20l100, mixture of 20 % ^{15}N and 100 % ^{15}N samples; 40l100, mixture of 40 % ^{15}N and 100 % ^{15}N samples; 0l60, mixture of ^{14}N and 60 % ^{15}N samples; 0l80, mixture of ^{14}N and 80 % ^{15}N samples.

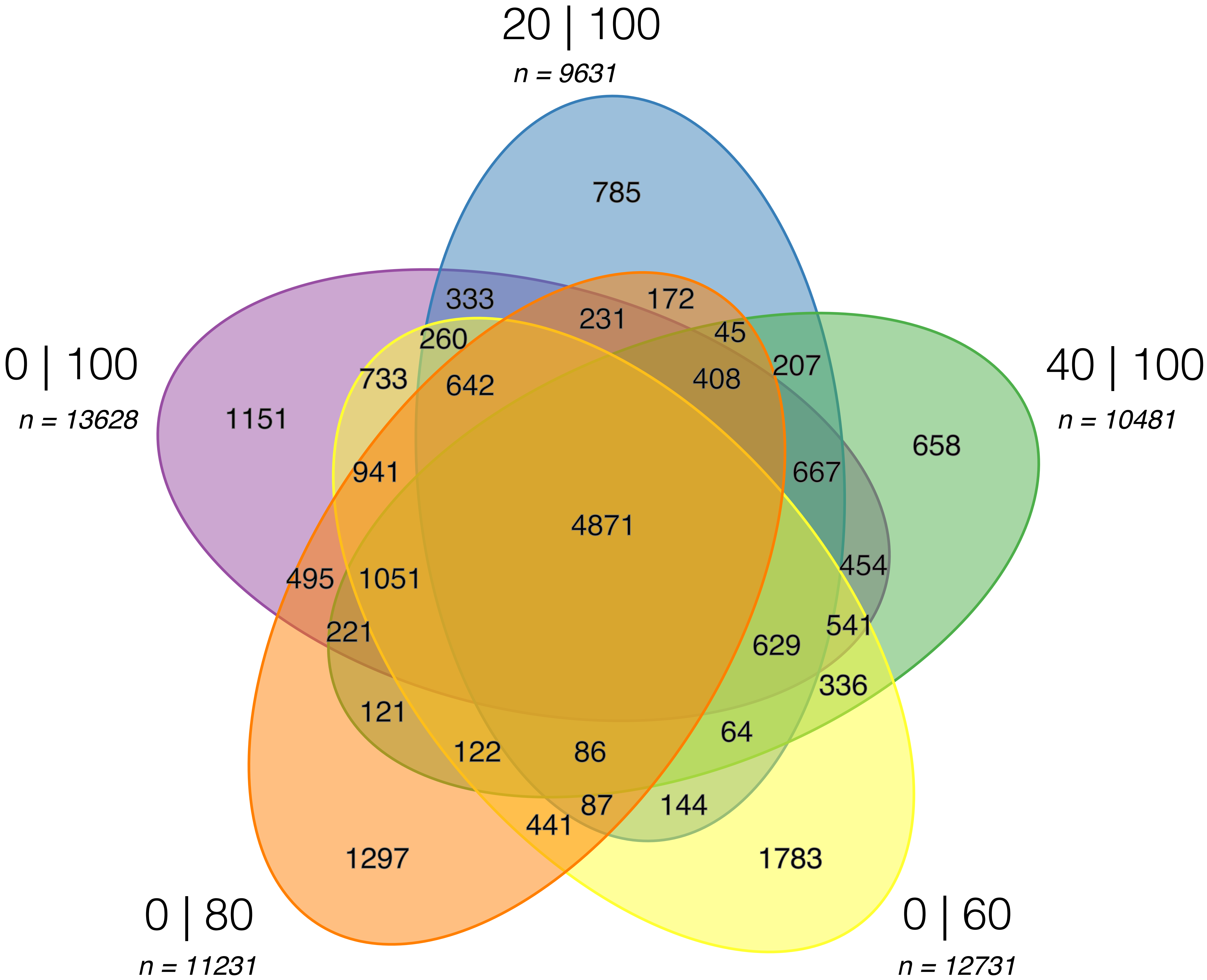

20 | 100
n = 9631
0 | 100
n = 13628
40 | 100
n = 10481
0 | 80
n = 11231
0 | 60
n = 12731
785
333
172
231
45
260
207
733
642
408
1151
658
941
667
4871
495
1051
454
221
541
629
336
121
64
122
86
87
144
441
1297
1783

Supplemental Figure 4

Peptide amounts (log2) for spiked-in-protein P02666

Peptide amounts (log2) for spiked-in-protein P02666 (Beta-Casein, *Bos taurus*) from master mix 3 for the DDA dataset using pyQms as quantification engine. Each sign represents the amount for one technical replicate of the indicated sample. X-axis shows the sample number, y-axis the determined log2 peptide amount. A liner regression was performed using Scipy (http://www.scipy.org/), the resulting function and the coefficient of determination is indicated in the figure. The peptide amount can accurately be determined over three orders of magnitude (1024 fold change) with a coefficient of determination of 0.989 in the presence of a complex background (human HEK293 cells).

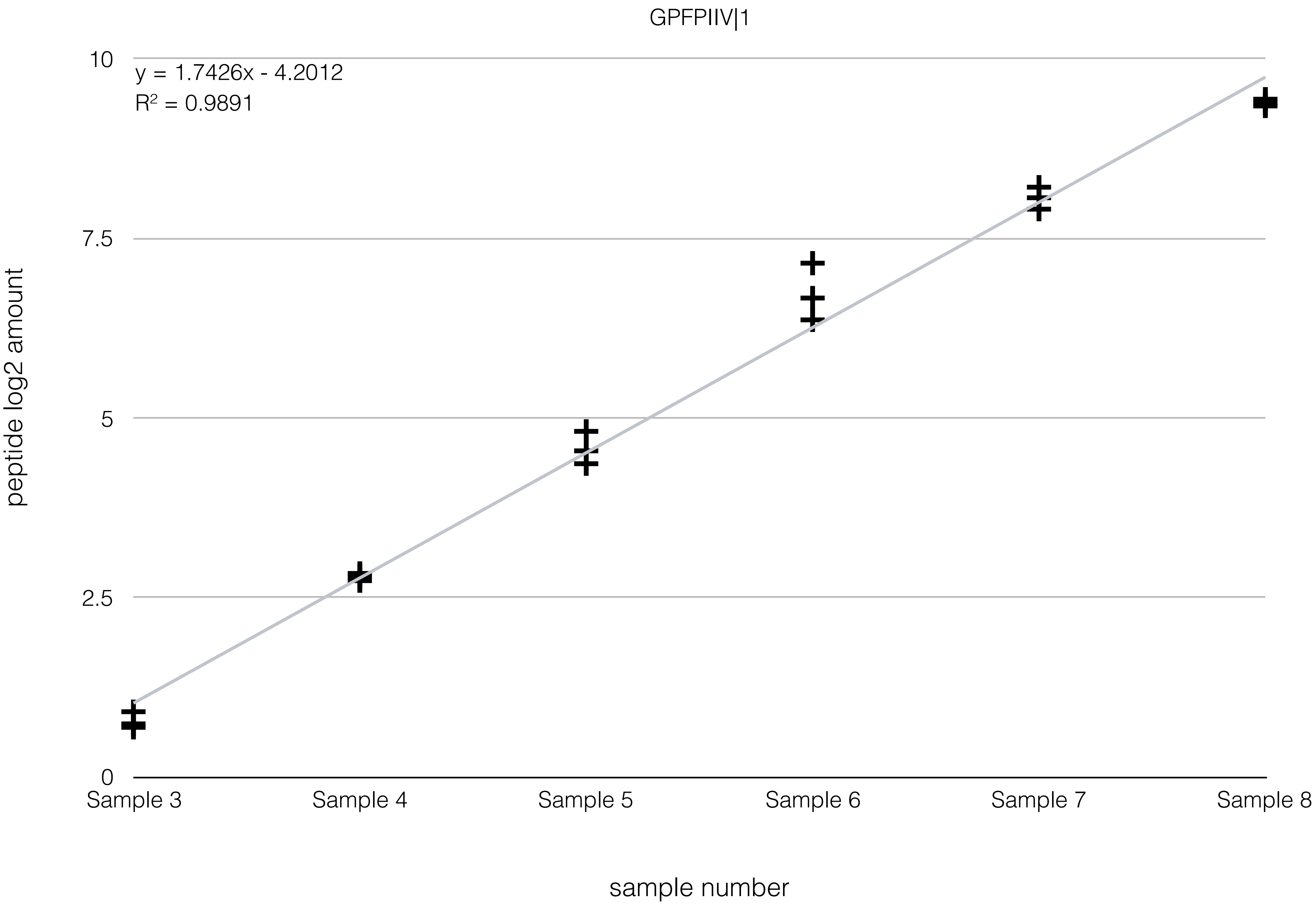
GPFPIIV|1
y = 1.7426x - 4.2012
R² = 0.9891
10
7.5
5
2.5
0
peptide log2 amount
Sample 3
Sample 4
Sample 5
Sample 6
Sample 7
Sample 8
sample number

Supplemental Figure 5

peptide intensities (log2) for spiked-in-protein P68082

Peptide amounts (log_2) for spiked-in-protein P68082 (Myoglobin, *Equus caballus*) from master mix 3 for the DDA data set from Bruderer et al. (2015) using pyQms as the quantification engine. Each sign represents the amount for one technical replicate of the indicated sample. X-axis shows the sample number, y-axis the determined log_2 peptide amount. Linear regressions were performed using Scipy (http://www.scipy.org/), the resulting function and the coefficient of determination are indicated in the figure. The peptide sequence and the charge state (separated with 'l') are shown above each sub plot.

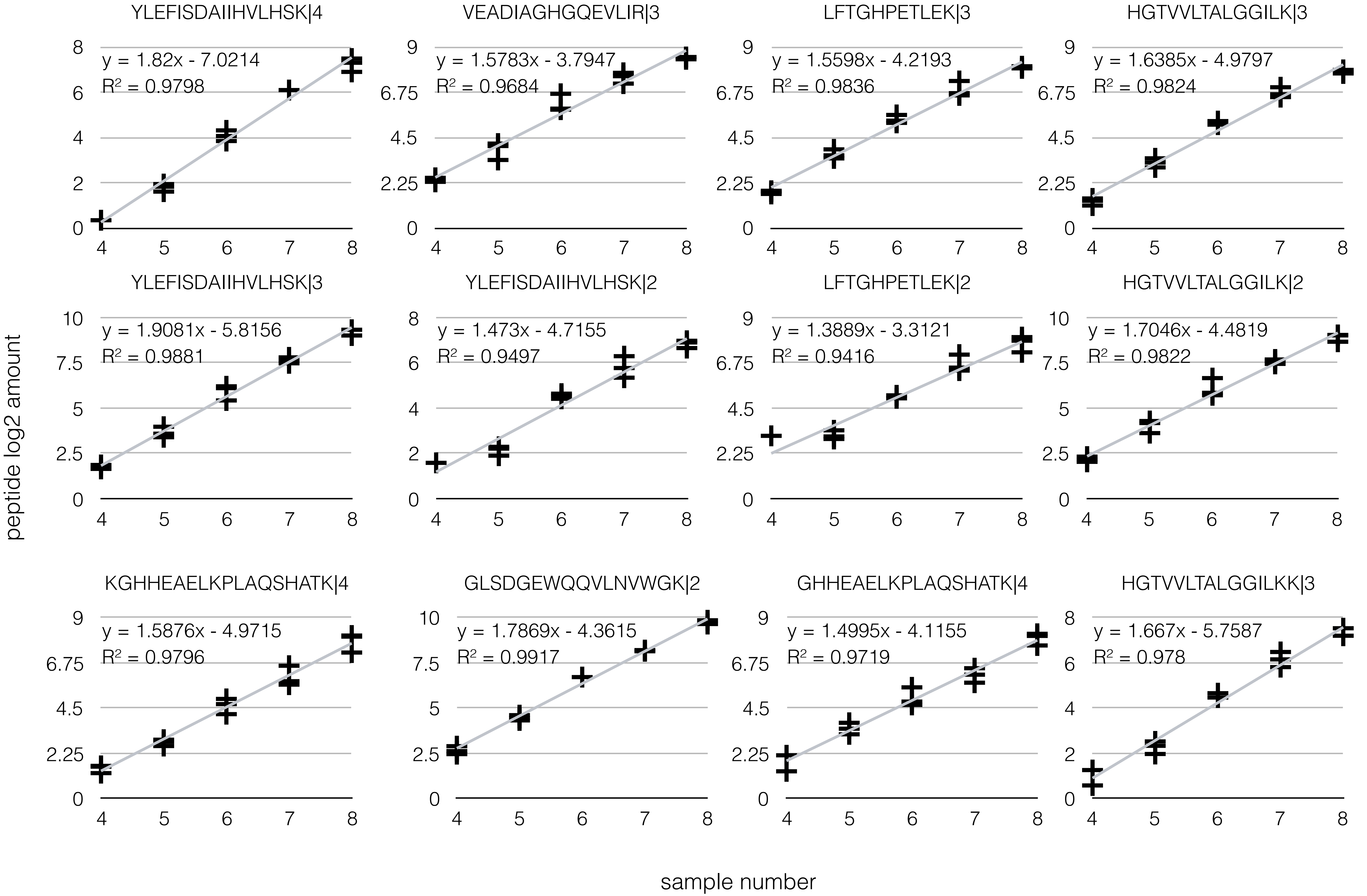
YLEFISDAIIHVLHSK|4
y = 1.82x - 7.0214
R² = 0.9798
VEADIAGHGQEVLIR|3
y = 1.5783x - 3.7947
R² = 0.9684
LFTGHPETLEK|3
y = 1.5598x - 4.2193
R² = 0.9836
HGTVVLTALGGILK|3
y = 1.6385x - 4.9797
R² = 0.9824
YLEFISDAIIHVLHSK|3
y = 1.9081x - 5.8156
R² = 0.9881
YLEFISDAIIHVLHSK|2
y = 1.473x - 4.7155
R² = 0.9497
LFTGHPETLEK|2
y = 1.3889x - 3.3121
R² = 0.9416
HGTVVLTALGGILK|2
y = 1.7046x - 4.4819
R² = 0.9822
KGHHEAELKPLAQSHATK|4
y = 1.5876x - 4.9715
R² = 0.9796
GLSDGEWQQVLNVWGK|2
y = 1.7869x - 4.3615
R² = 0.9917
GHHEAELKPLAQSHATK|4
y = 1.4995x - 4.1155
R² = 0.9719
HGTVVLTALGGILKK|3
y = 1.667x - 5.7587
R² = 0.978
peptide log2 amount
sample number

Supplemental Figure 6

Peptide amount correlation of spiked-in-proteins of pyQms vs. MaxQuant

Peptide amount correlation of pyQms against MaxQuant (n=3,673 peptide-charge combinations). Peptide amount correlation of spiked-in-proteins from the Bruderer *et al.* (2015) DDA data set. The x-axis represents the pyQms peptide log2 intensity and the y-axis the MaxQuant peptide log2 intensity. The heat represent the counts in the intensity bin. Amounts were rounded to 1 decimal place. Single counts in bins are shown in grey squares. The coefficient of determination was determined using the Python Scipy linear regression function (http://www.scipy.org/) on non-log2 transformed amounts and is shown in the upper right corner. Samples were not intensity aligned.

R^2=0.986
n=3673

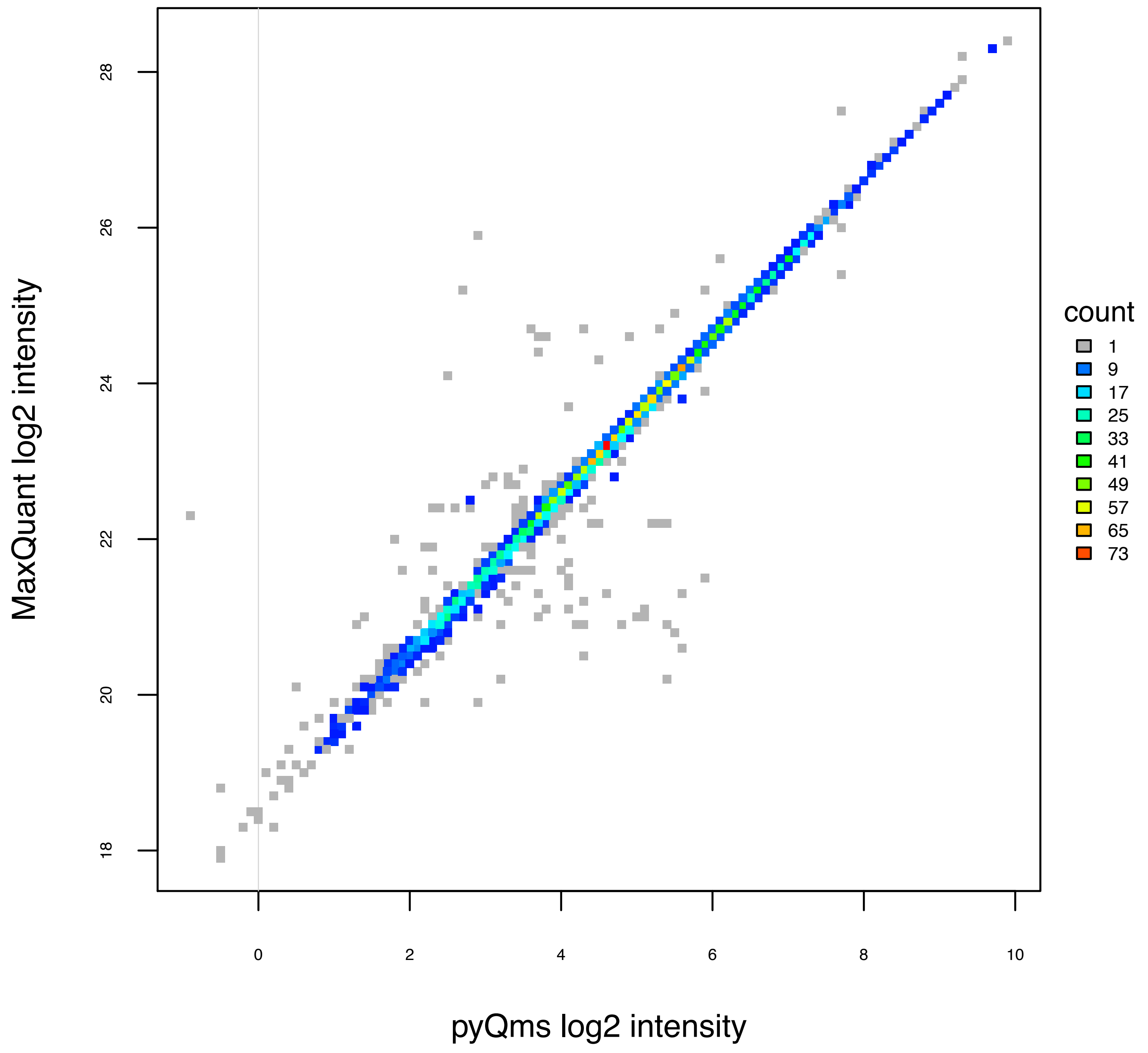

Supplemental Figure 7

Peptide amount correlation of background and spiked-in-proteins of pyQms vs. MaxQuant

Peptide amount correlation of pyQms vs. MaxQuant (n=247,228 peptide-charge combinations). Peptide amount correlation of human background proteins (HEK293 cells) from the DDA dataset of Bruderer *et al.* (2015). Shown are the log2 peptide amounts, x-axis represents pyQms amounts and y-axis MaxQuant amounts. The heat represents the counts in the intensity bin. Counts ≤ 0.1% of the maximum count are shown in grey. R^2 was determined using the Python Scipy linear regression function (http://www.scipy.org/) on non-log2 transformed amounts and is shown in the upper right corner. Samples were not intensity aligned.

$R^2=0.983$
n=247228

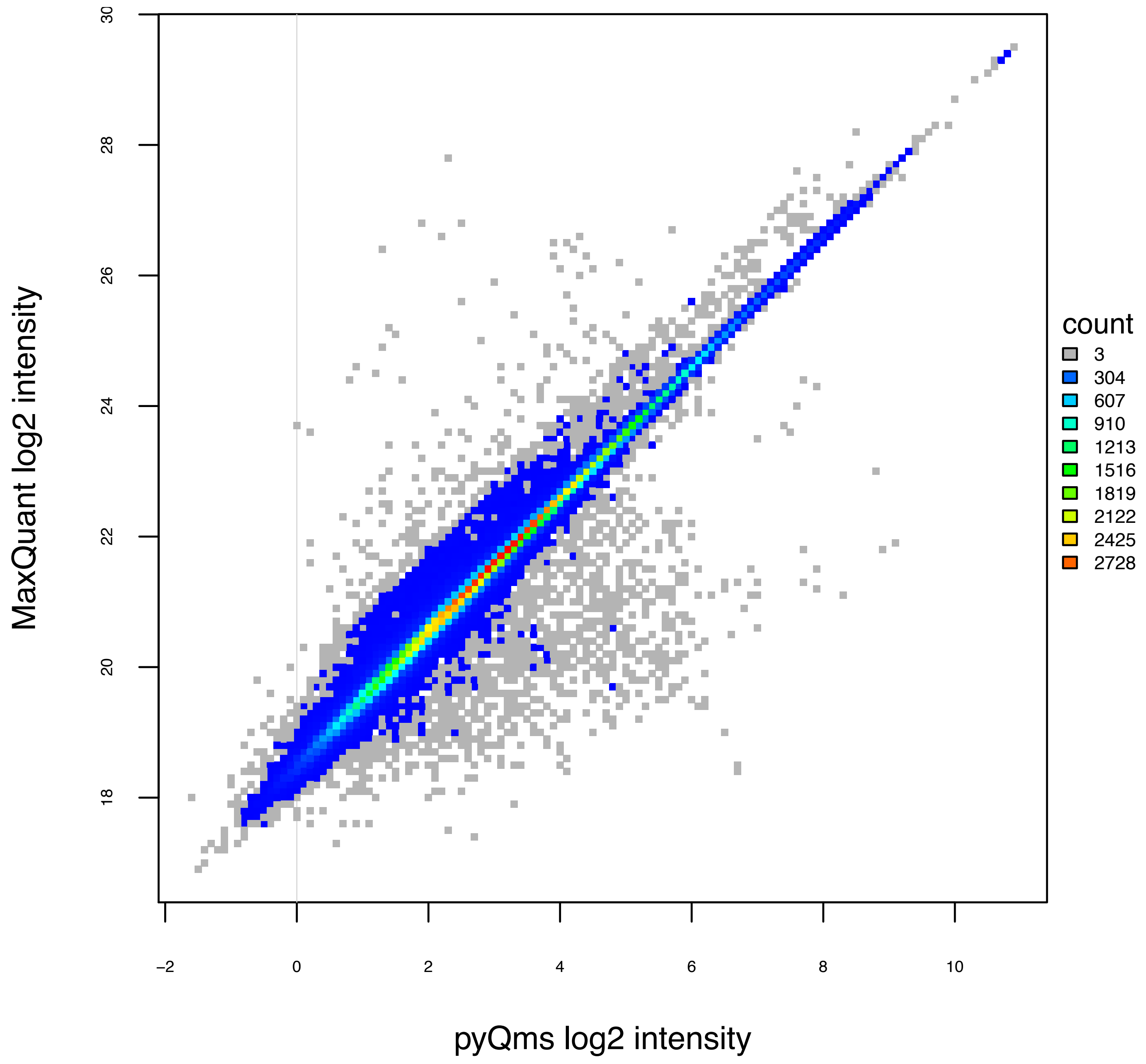

Supplemental Figure 8

Ratio evaluation summary on protein level

Stacked bar plot for relative occurrences of the p-values of all 12 single spiked-in-proteins for each protein separate of the data set from Bruderer *et al.* (2015). Calculated p-values for peptide ratios on protein level were counted for DDA and DIA mode separately and for amounts determined by pyQms (for DDA and DIA) as well as MaxQuant (bruderer_dda, DDA) and Spectronaut (bruderer_hrm, DIA). Counts are displayed relatively to the maximum count of possible peptides ratios. Legend: x-axis, combination of master mix, protein, quantification software and data acquisition mode (grouped according to master mix group); y-axis, relative occurrence of p-value bin. t-test p-value legend, blue: not significant different, green: p-value <= 0.05, yellow: p-value <= 0.01, red: p-value <= 0.001.

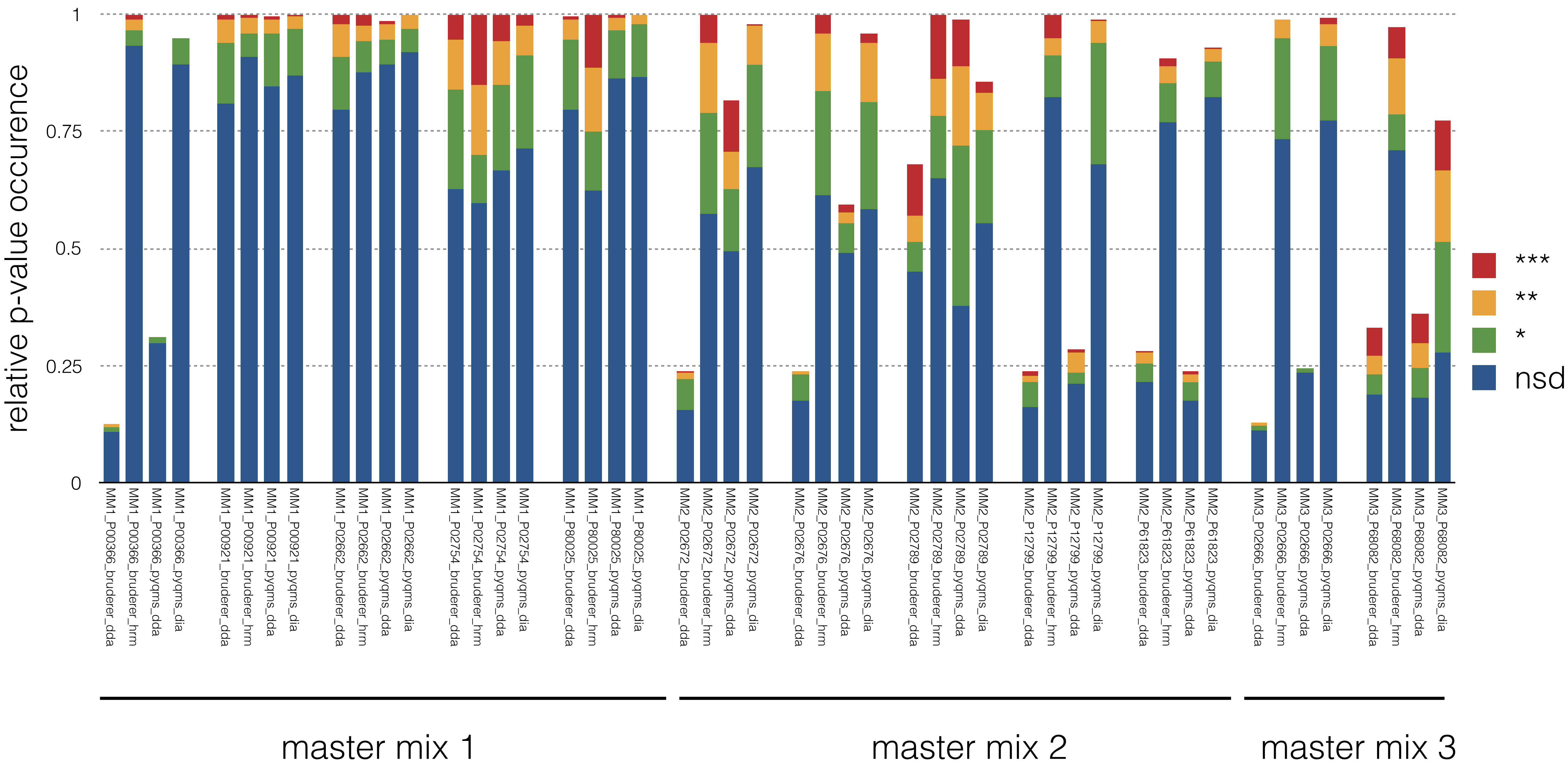

relative p-value occurence
1
0.75
0.5
0.25
0
***
**
*
nsd
MM1_P00366_bruderer_dda
MM1_P00366_bruderer_hrm
MM1_P00366_pyqms_dda
MM1_P00366_pyqms_dia
MM1_P00921_bruderer_dda
MM1_P00921_bruderer_hrm
MM1_P00921_pyqms_dda
MM1_P00921_pyqms_dia
MM1_P02662_bruderer_dda
MM1_P02662_bruderer_hrm
MM1_P02662_pyqms_dda
MM1_P02662_pyqms_dia
MM1_P02754_bruderer_dda
MM1_P02754_bruderer_hrm
MM1_P02754_pyqms_dda
MM1_P02754_pyqms_dia
MM1_P80025_bruderer_dda
MM1_P80025_bruderer_hrm
MM1_P80025_pyqms_dda
MM1_P80025_pyqms_dia
MM2_P02672_bruderer_dda
MM2_P02672_bruderer_hrm
MM2_P02672_pyqms_dda
MM2_P02672_pyqms_dia
MM2_P02676_bruderer_dda
MM2_P02676_bruderer_hrm
MM2_P02676_pyqms_dda
MM2_P02676_pyqms_dia
MM2_P02789_bruderer_dda
MM2_P02789_bruderer_hrm
MM2_P02789_pyqms_dda
MM2_P02789_pyqms_dia
MM2_P12799_bruderer_dda
MM2_P12799_bruderer_hrm
MM2_P12799_pyqms_dda
MM2_P12799_pyqms_dia
MM2_P61823_bruderer_dda
MM2_P61823_bruderer_hrm
MM2_P61823_pyqms_dda
MM2_P61823_pyqms_dia
MM3_P02666_bruderer_dda
MM3_P02666_bruderer_hrm
MM3_P02666_pyqms_dda
MM3_P02666_pyqms_dia
MM3_P68082_bruderer_dda
MM3_P68082_bruderer_hrm
MM3_P68082_pyqms_dda
MM3_P68082_pyqms_dia
master mix 1
master mix 2
master mix 3

Supplemental Figure 9

Visual label-free ratio evaluation

Heat maps of label-free ratio evaluation (t-test p-value) of 12 spiked-in-proteins (A-L) of the data set from Bruderer *et al.* (2015). Results from pyQms are compared against MaxQuant and Spectronaut. Numbers in the bins show the number of peptide charge combination ratios (DDA) or peptide ratios (DIA) used for the t-test. Each bin reflects a p-value calculated for peptide ratios between the indicated sample and technical replicate (total 24) on the x and y-axis. blue: not significant different, green: p-value <= 0.05, yellow: p-value <= 0.01, red: p-value <= 0.001. Histograms on the top and right indicate the relative spiked-in amount for the samples; x-axes, sample; y-axes, relative spiked-in protein concentration.

Proteins A-L panel 1: The upper half shows the t-test ratio evaluation results for the DDA data using pyQms for quantification. The lower half shows the t-test ratio evaluation results for the DIA data using pyQms for quantification. Proteins A-L panel 2: The upper half shows the t-test ratio evaluation results for the DDA data using pyQms for quantification. The lower half shows the t-test ratio evaluation results for the DDA data using MaxQuant for quantification (using results from Bruderer *et al.* 2015). Proteins A-L panel 3: The upper half shows the t-test ratio evaluation results for the DIA data using pyQms for quantification. The lower half shows the t-test ratio evaluation results for the DIA data using Spectronaut for quantification (using results from Bruderer *et al.* 2015).

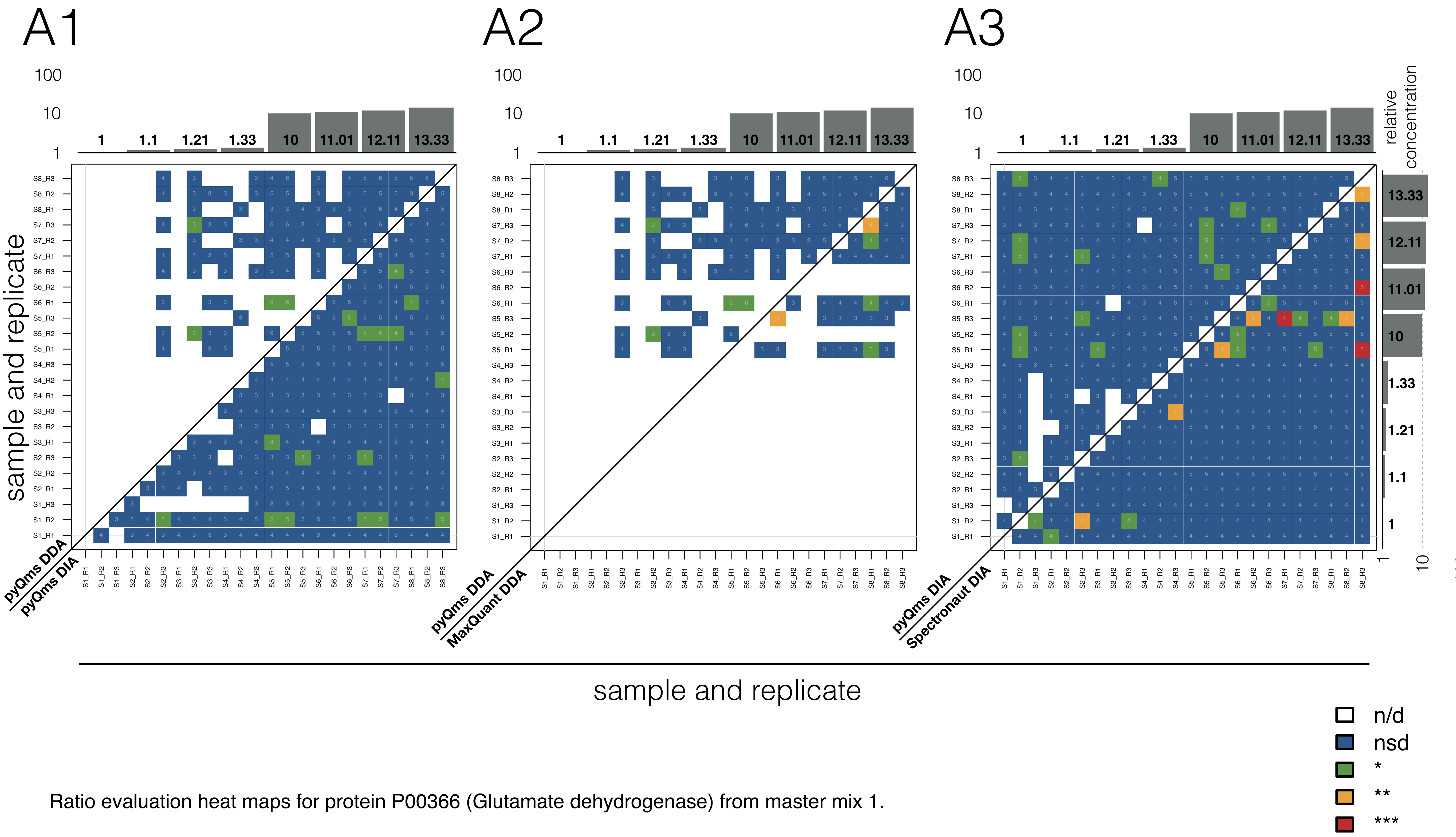

Ratio evaluation heat maps for protein P00366 (Glutamate dehydrogenase) from master mix 1.

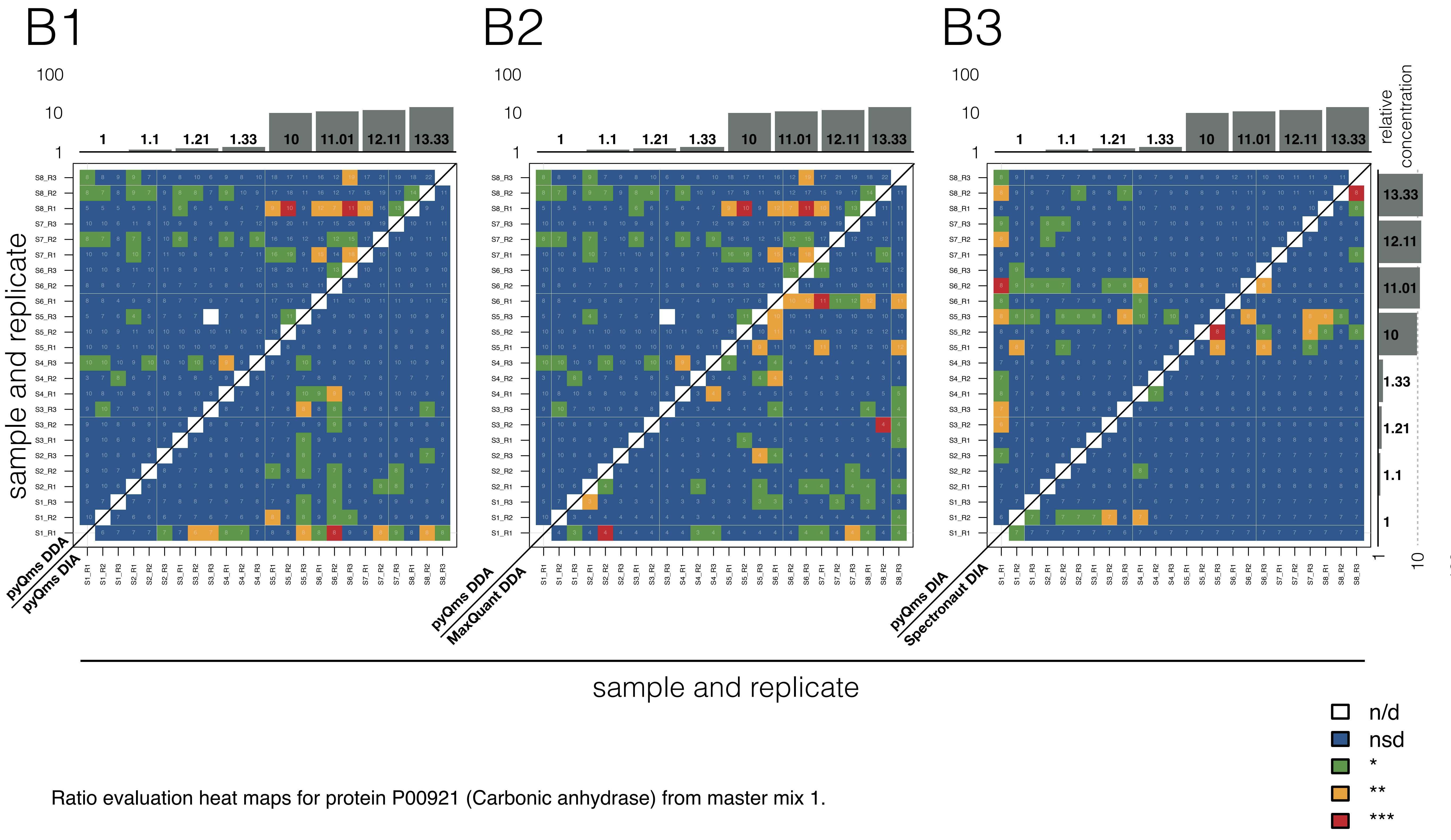

Ratio evaluation heat maps for protein P00921 (Carbonic anhydrase) from master mix 1.

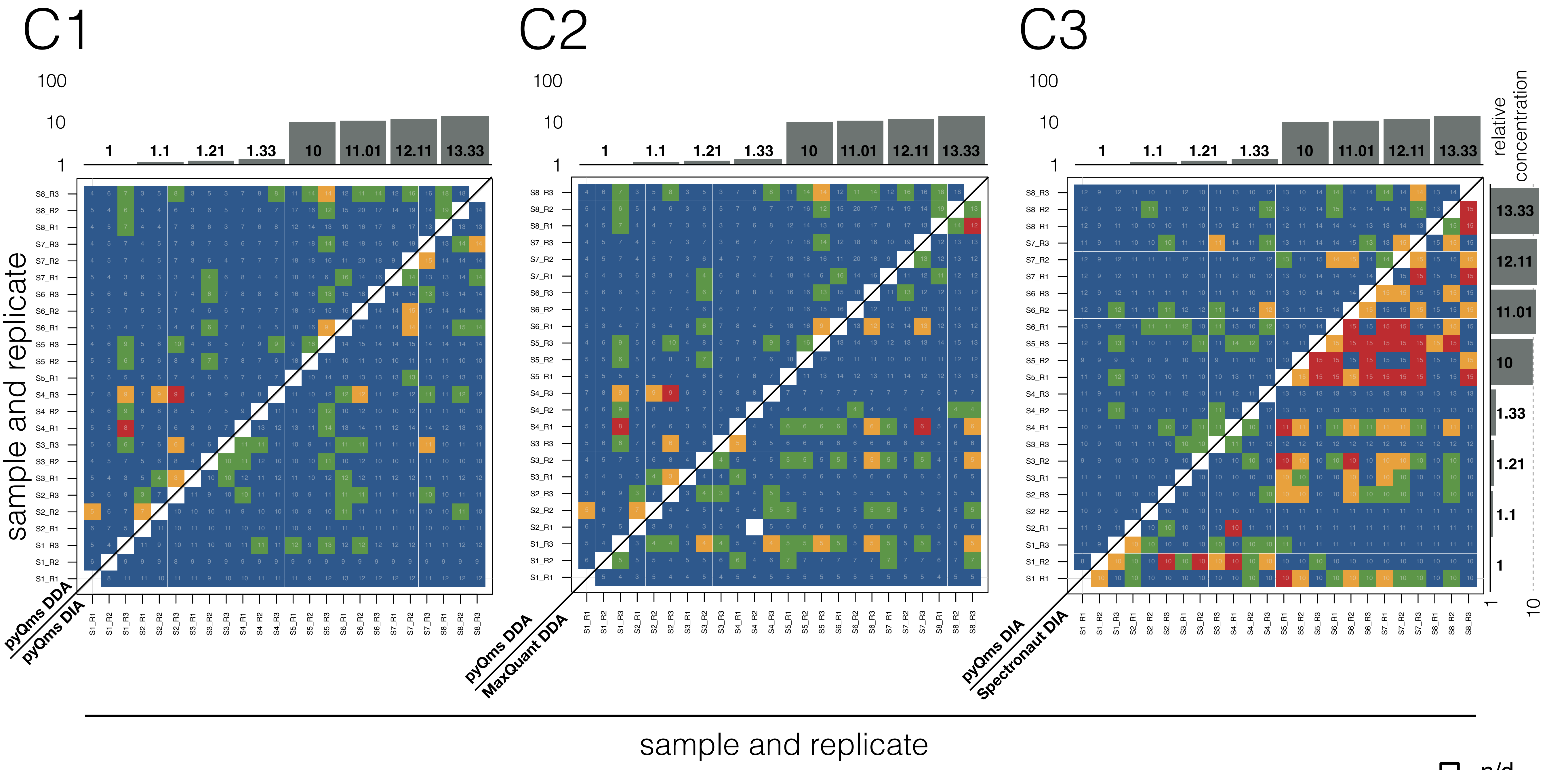

Ratio evaluation heat maps for protein P80025 (Lactoperoxidase) from master mix 1.

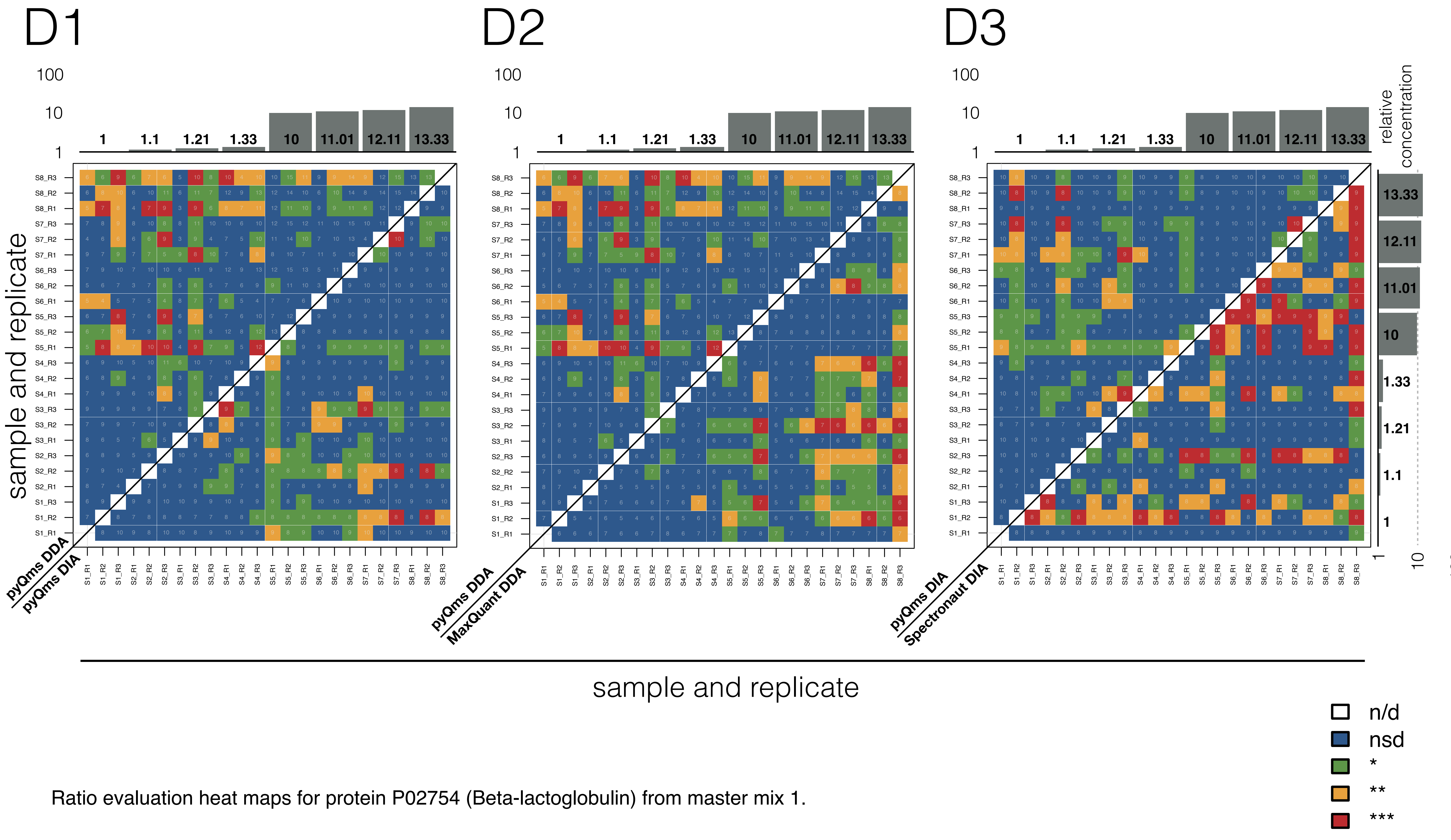

Ratio evaluation heat maps for protein P02754 (Beta-lactoglobulin) from master mix 1.

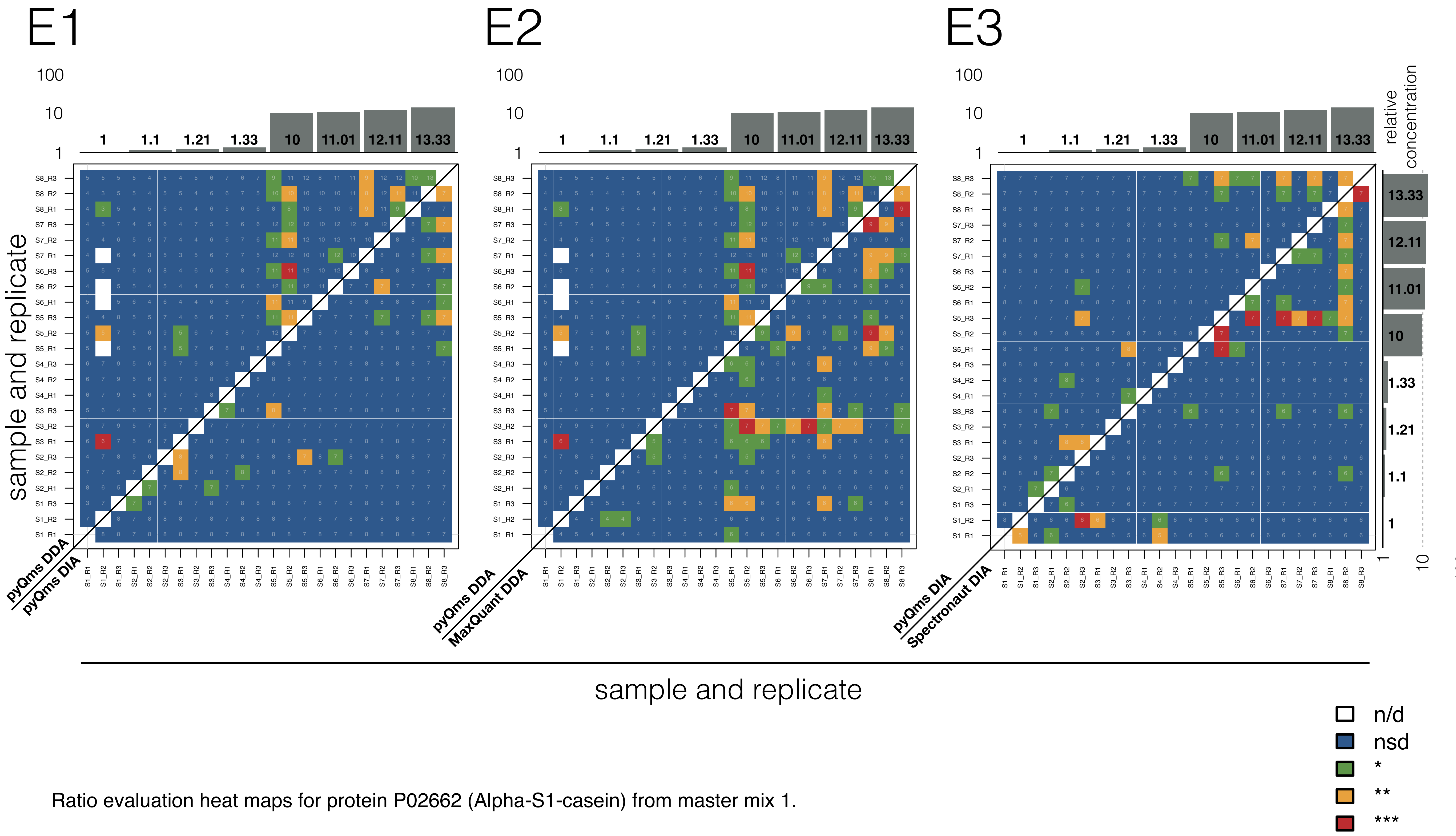

Ratio evaluation heat maps for protein P02662 (Alpha-S1-casein) from master mix 1.

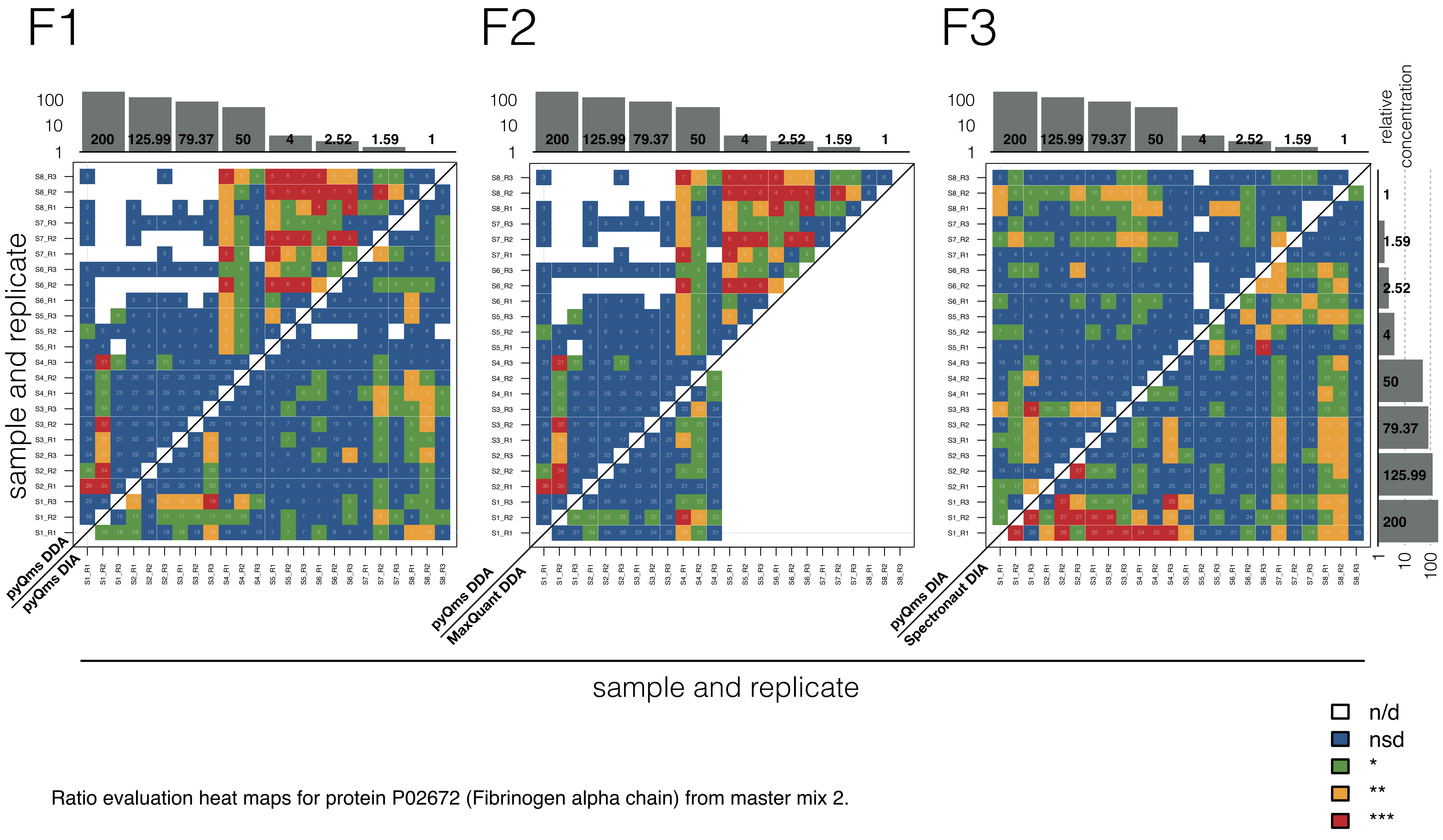

Ratio evaluation heat maps for protein P02672 (Fibrinogen alpha chain) from master mix 2.

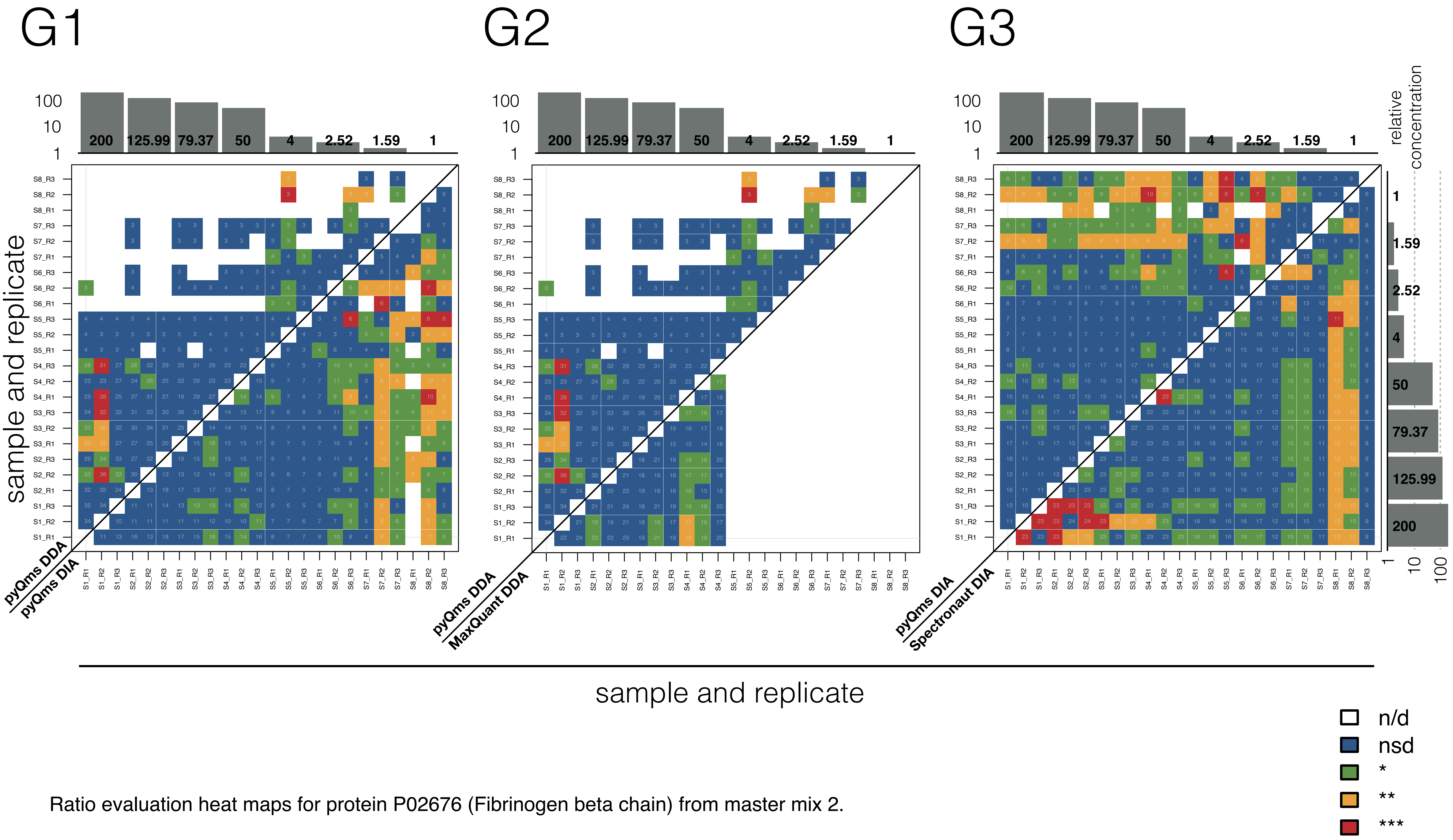

Ratio evaluation heat maps for protein P02676 (Fibrinogen beta chain) from master mix 2.

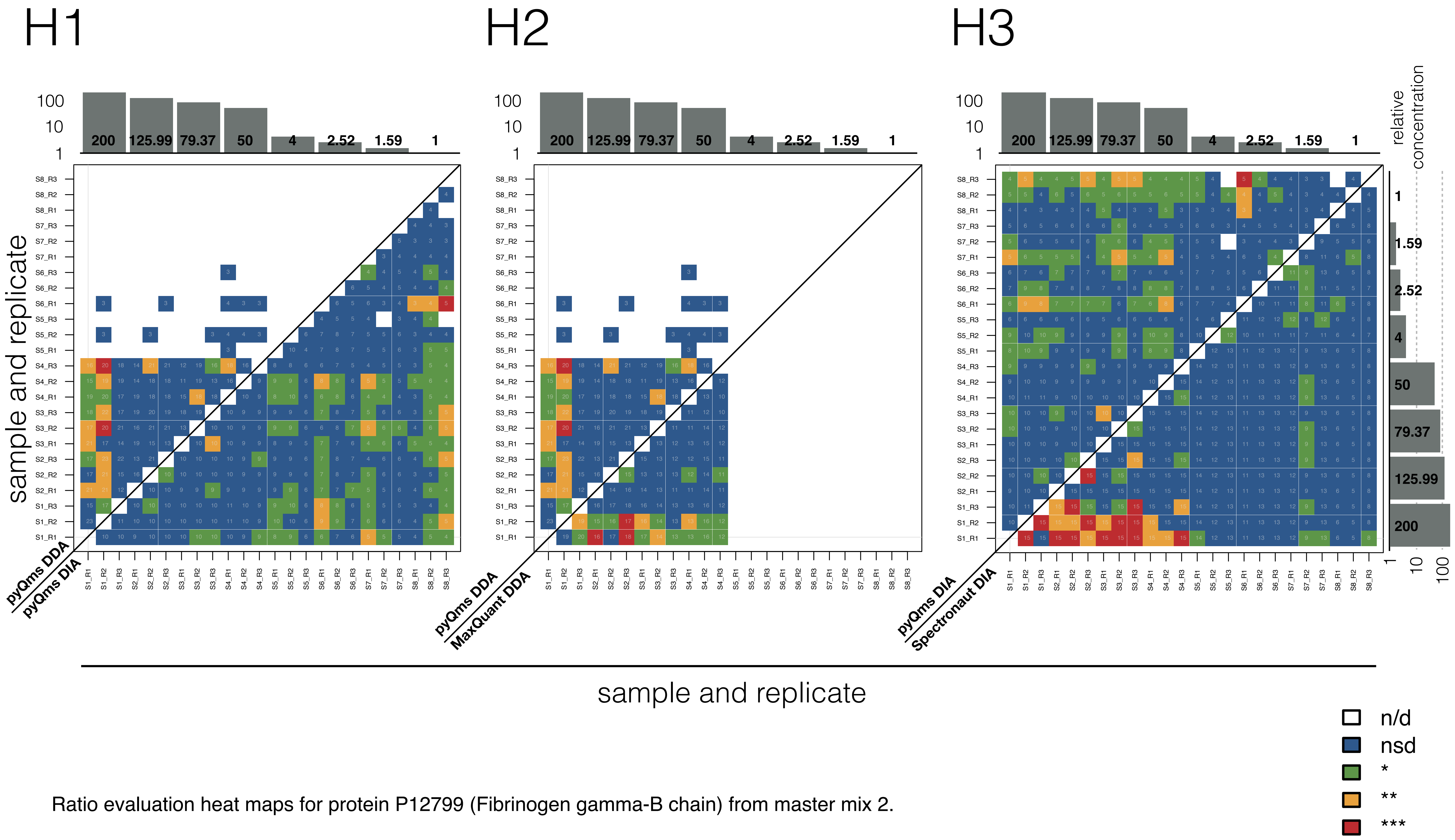

Ratio evaluation heat maps for protein P12799 (Fibrinogen gamma-B chain) from master mix 2.

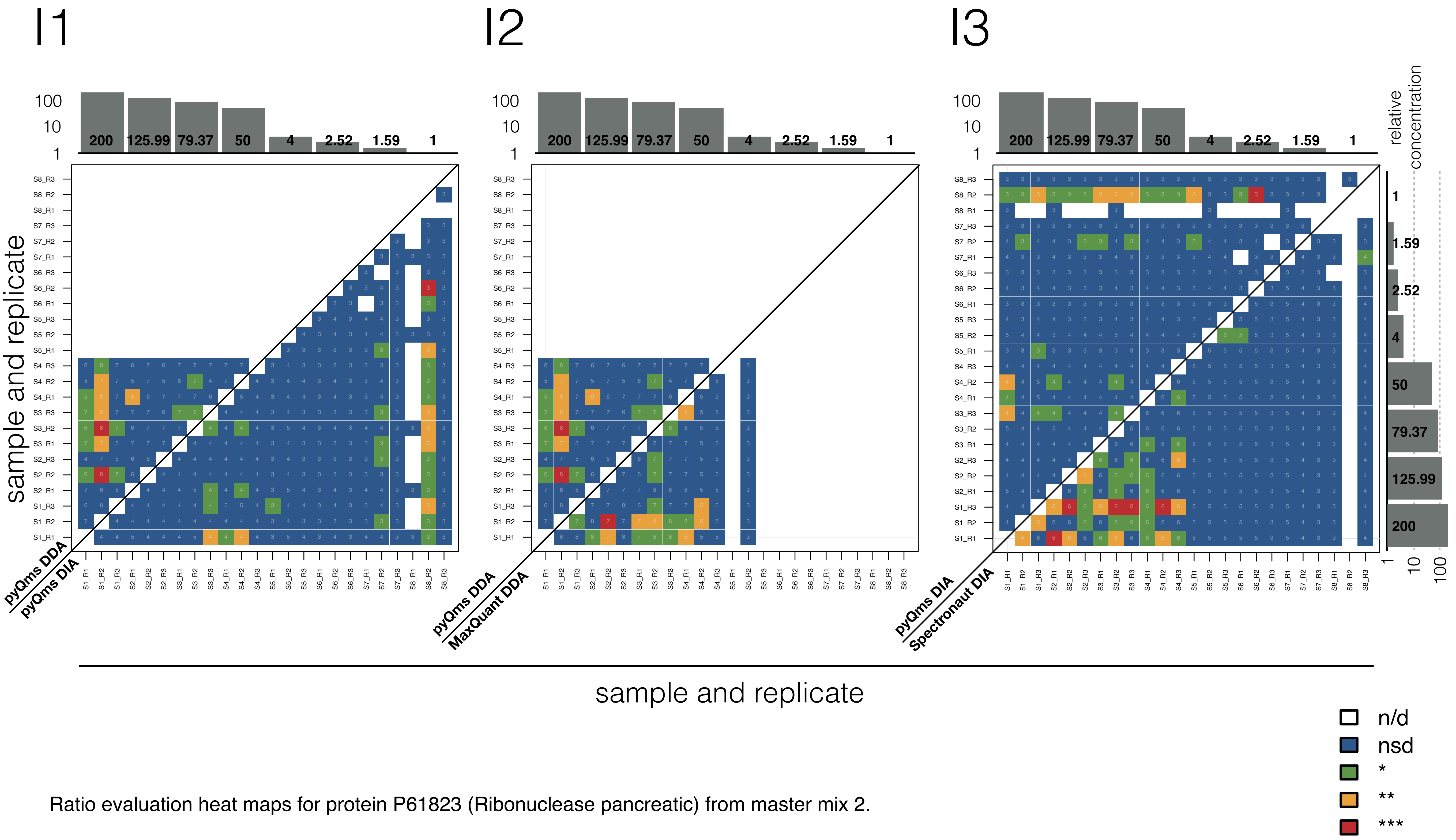

Ratio evaluation heat maps for protein P61823 (Ribonuclease pancreatic) from master mix 2.

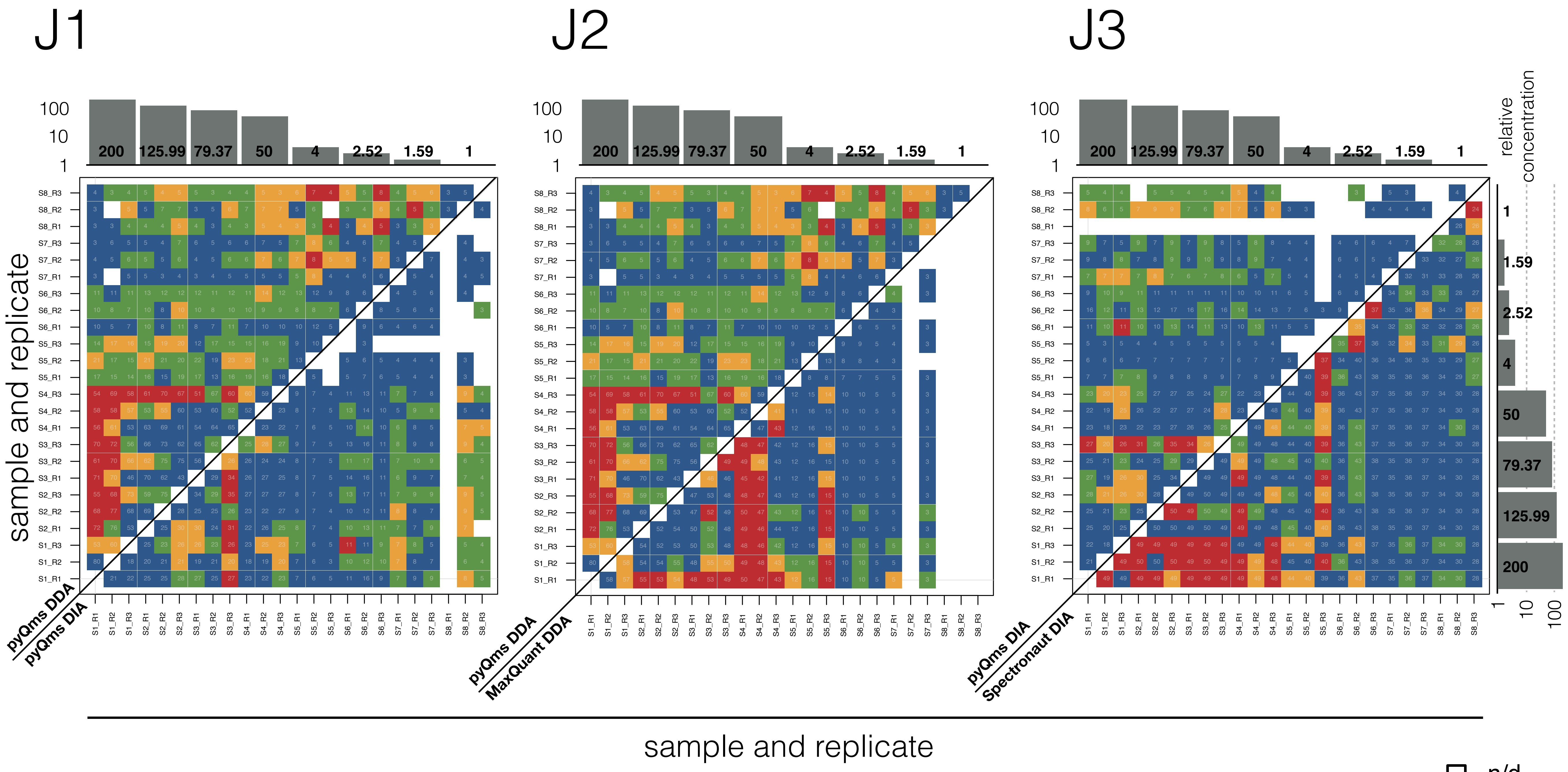

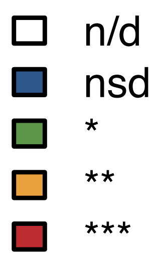

Ratio evaluation heat maps for protein P02789 (Ovotransferrin) from master mix 2.

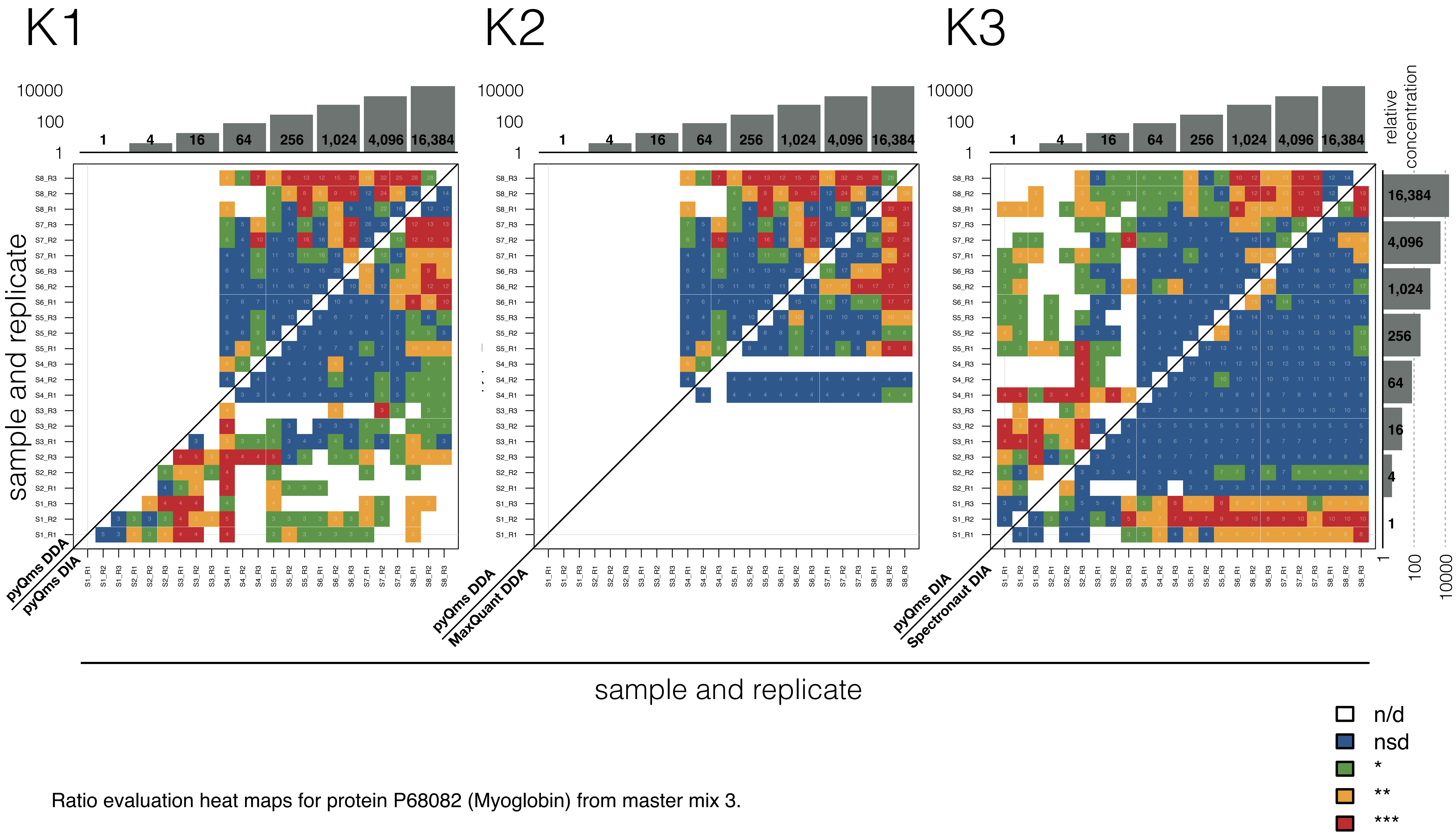

Ratio evaluation heat maps for protein P68082 (Myoglobin) from master mix 3.

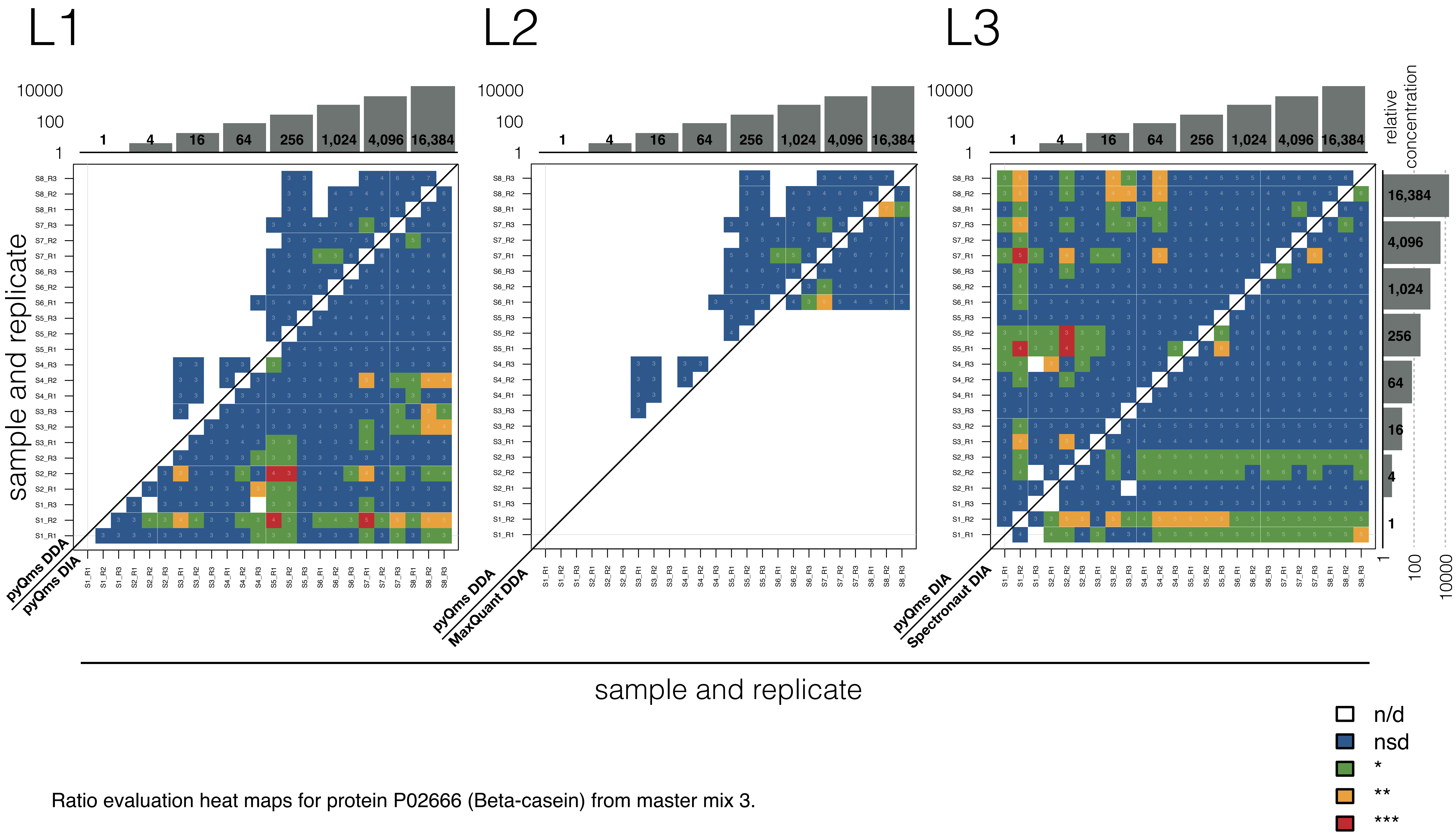

Ratio evaluation heat maps for protein P02666 (Beta-casein) from master mix 3.

Supplemental Figure 10

Ratio evaluation summary on master mix level.

Stacked bar plot for relative occurrences of the p-values (binned) of all 12 spiked-in-proteins on master mix level. Calculated p-values for peptide ratios on protein level were counted and binned for DDA and DIA mode separately and for amount determined by pyQms (for DDA and DIA) as well as MaxQuant (bruderer_dda, DDA) and Spectronaut (bruderer_hrm, DIA). Counts are displayed relatively to the maximum count of possible ratios (numbers on bars). Legend: x-axis, combination of master mix, quantification engine and data acquisition mode (grouped according to master mix); y-axis, relative occurrence of p-value (binned); t-test p-value legend, blue: not significant different, green: p-value <= 0.05, yellow: p-value <= 0.01, red: p-value <= 0.001.

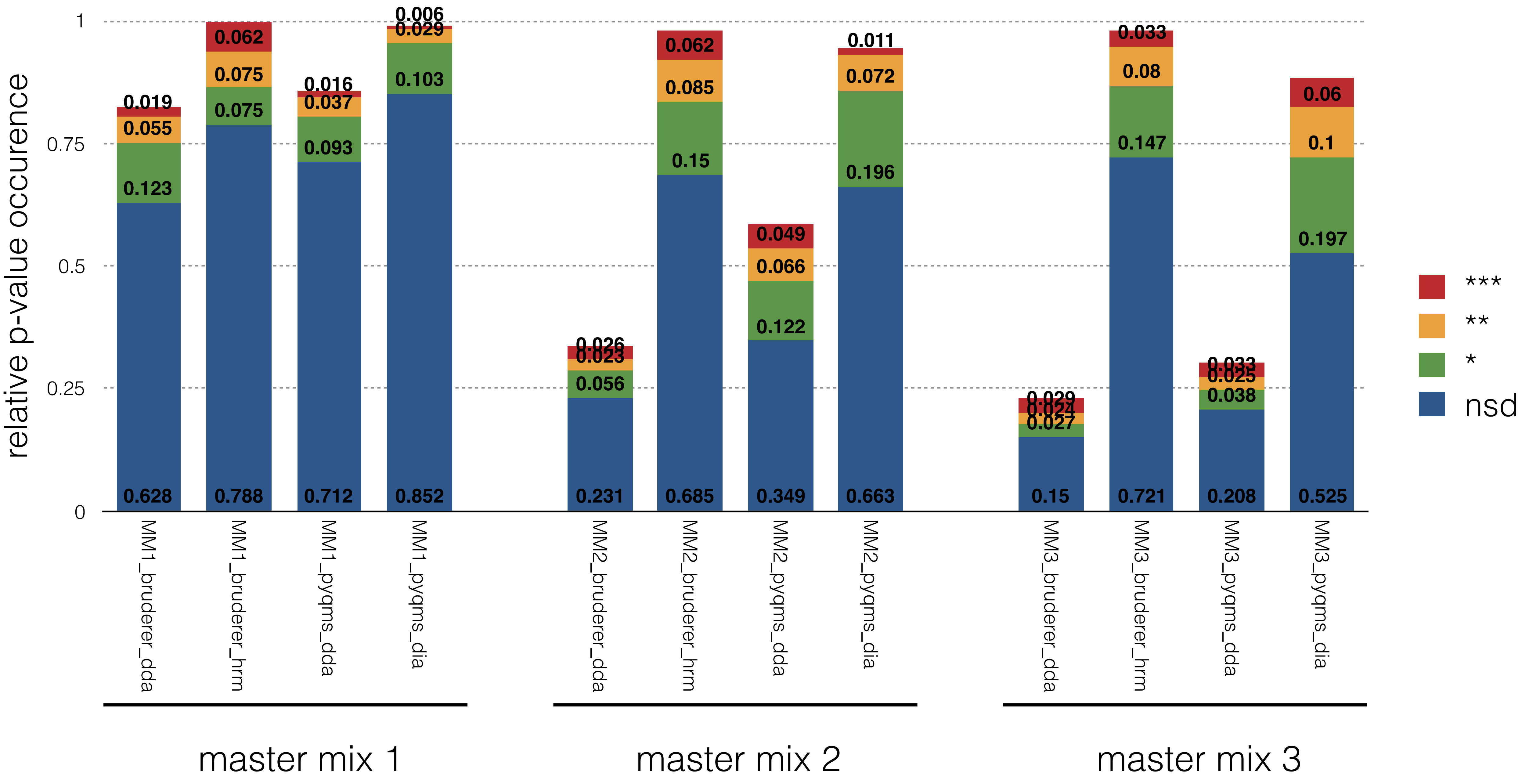
relative p-value occurence
1
0.75
0.5
0.25
0
0.628
0.123
0.055
0.019
0.788
0.075
0.075
0.062
0.712
0.093
0.037
0.016
0.852
0.103
0.029
0.006
0.231
0.056
0.023
0.026
0.685
0.15
0.085
0.062
0.349
0.122
0.066
0.049
0.663
0.196
0.072
0.011
0.15
0.027
0.024
0.029
0.721
0.147
0.08
0.033
0.208
0.038
0.025
0.033
0.525
0.197
0.1
0.06
MM1_bruderer_dda
MM1_bruderer_hrm
MM1_pyqms_dda
MM1_pyqms_dia
MM2_bruderer_dda
MM2_bruderer_hrm
MM2_pyqms_dda
MM2_pyqms_dia
MM3_bruderer_dda
MM3_bruderer_hrm
MM3_pyqms_dda
MM3_pyqms_dia
master mix 1
master mix 2
master mix 3
***
**
*
nsd

Supplemental Figure 11

Quantification engine comparison

The quantification engines MaxQuant, Spectronaut and pyQms were compared. The absolute deviation of log2 peptide ratios from the ground truth was assessed for all spiked-in proteins in the data set of Bruderer *et al.* (2015). The observed label-free log2 peptide ratios between all sample combinations were calculated. The deviations between the observed and expected (ground truth) ratios (log2) was calculated and are displayed as density plots and as box plots in the inset. Density plot: x-axis, absolute deviation of log2 peptide ratios from log2 ground truth ratio; y-axis, density. Box plot: x-axis, quantification engine and data acquisition mode combination, y-axis, absolute deviation of log2 peptide ratios from log2 ground truth ratio. The legend shows the total number of peptide ratios compared (n) and the x-position of the maximum density (max@). MaxQuant: red, n=17520; Spectronaut: light green, n=48145; pyQms (DDA): green, n=24511; pyQms (DIA): blue, n=23741. * Please note, that for the pyQms DIA ratio evaluation a simple, preliminary algorithm was used to select the fragment ions used for quantification. A fair comparison to Spectronaut would be only possible, if all ions selected for quantification by Spectronaut would have been available.

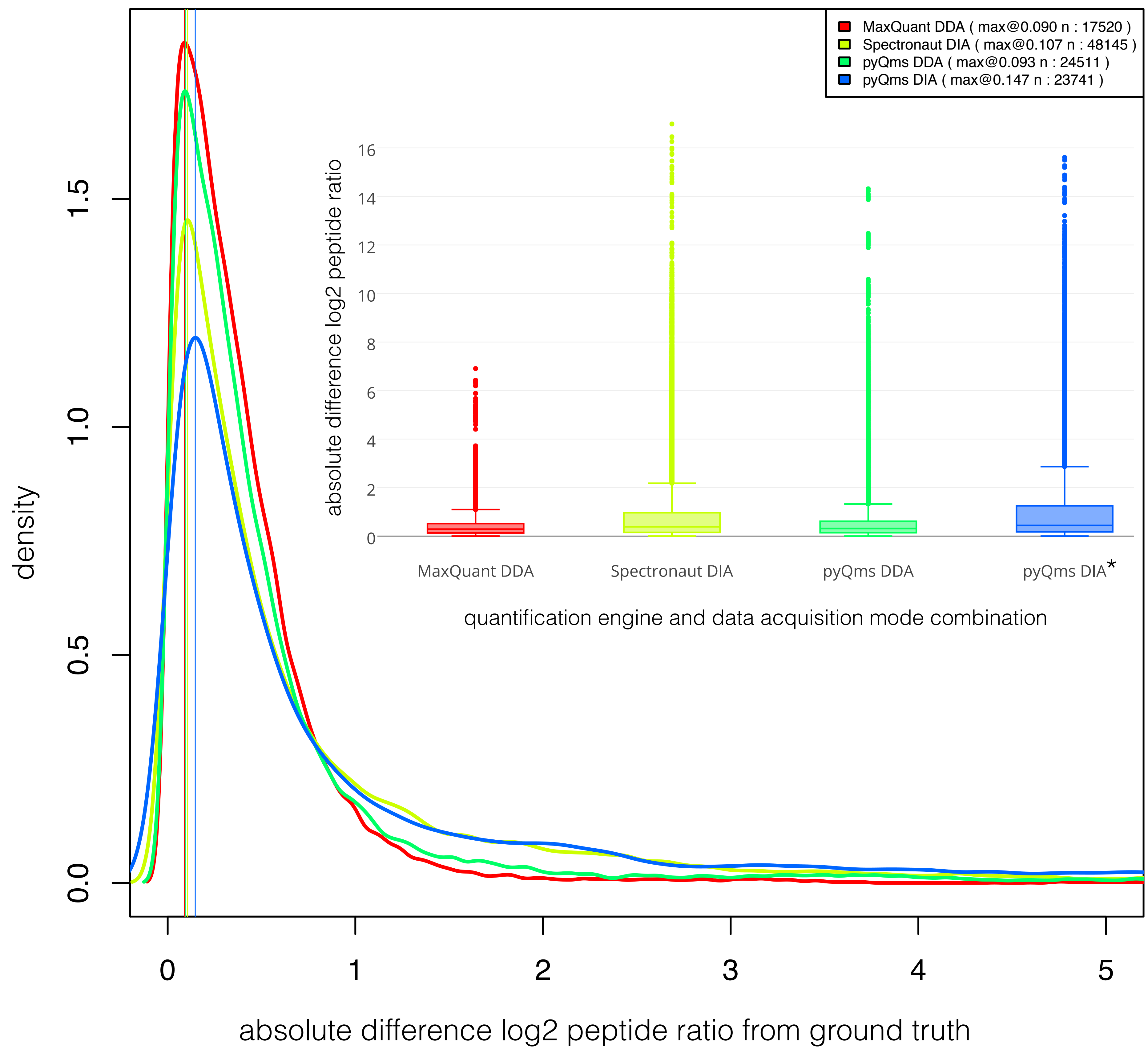

MaxQuant DDA (max@0.090 n : 17520)
Spectronaut DIA (max@0.107 n : 48145)
pyQms DDA (max@0.093 n : 24511)
pyQms DIA (max@0.147 n : 23741)
density
0.0
0.5
1.0
1.5
0
1
2
3
4
5
absolute difference log2 peptide ratio from ground truth
absolute difference log2 peptide ratio
0
2
4
6
8
10
12
14
16
MaxQuant DDA
Spectronaut DIA
pyQms DDA
pyQms DIA*
quantification engine and data acquisition mode combination

Supplemental Figure 12

Visual partially labeled gold standard data set evaluation

A, C, E, and G; heat map showing the matched labelling percentiles combined for each 7 LC-MS/MS runs. Whole cell samples were mixed on equal chlorophyll level and measured via LC-MS/MS. All identified peptides (19,976 peptide sequences, PEP <= 0.01) from all samples were quantified in all LC-MS/MS runs in 5 charge states and labelling percentiles ranging from 0 to 99 % in 1% steps. Heat maps correspond to Fig. 3D.

B, D, F and H; same as heat maps A, C, E and G but only quantifications are considered which appear in stringent determined retention time windows after retention time alignment. Heat maps correspond to Figure 3G.

A and B, 0 and 100% mixture; C and D, 0 and 80% mixture; E and F, 20 and 100% mixture; G and H, 40 and 100% mixture. The x-axes of the heat maps represents the labelling percentile bin (0 - 100%; 0-1), y-axes represents the mScore of the matches (0.5 - 1). The heat represents the summed up intensities of all peptide matches in all MS1 spectra in the respective labelling percentile – mScore bin. Histograms at axes indicate the total count in the respective column or row. Legend bars at the bottom indicate the color to total intensity relation ranging from blue (minimum intensity) to red/yellow (medium intensity) to white (maximum intensity). The intensity thresholds for the heat bins is indicated.

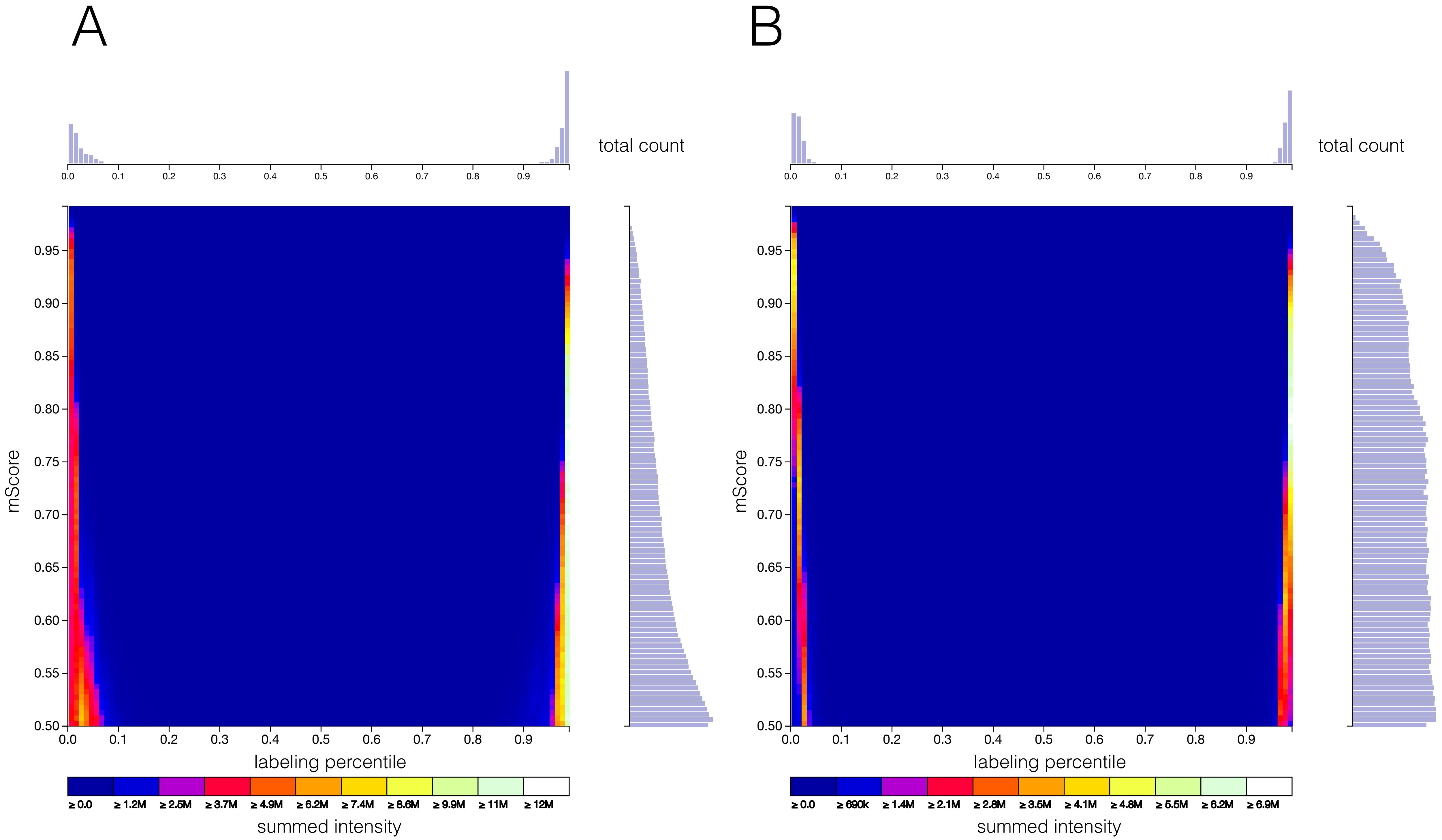

Heats maps for the 0 and 100% mixture of pulse chase gold standard data set. A, all peptides were quantified in all spectra. B, only quantifications of peptides were allowed for spectra which appeared in stringent determined peptide specific retention time windows.

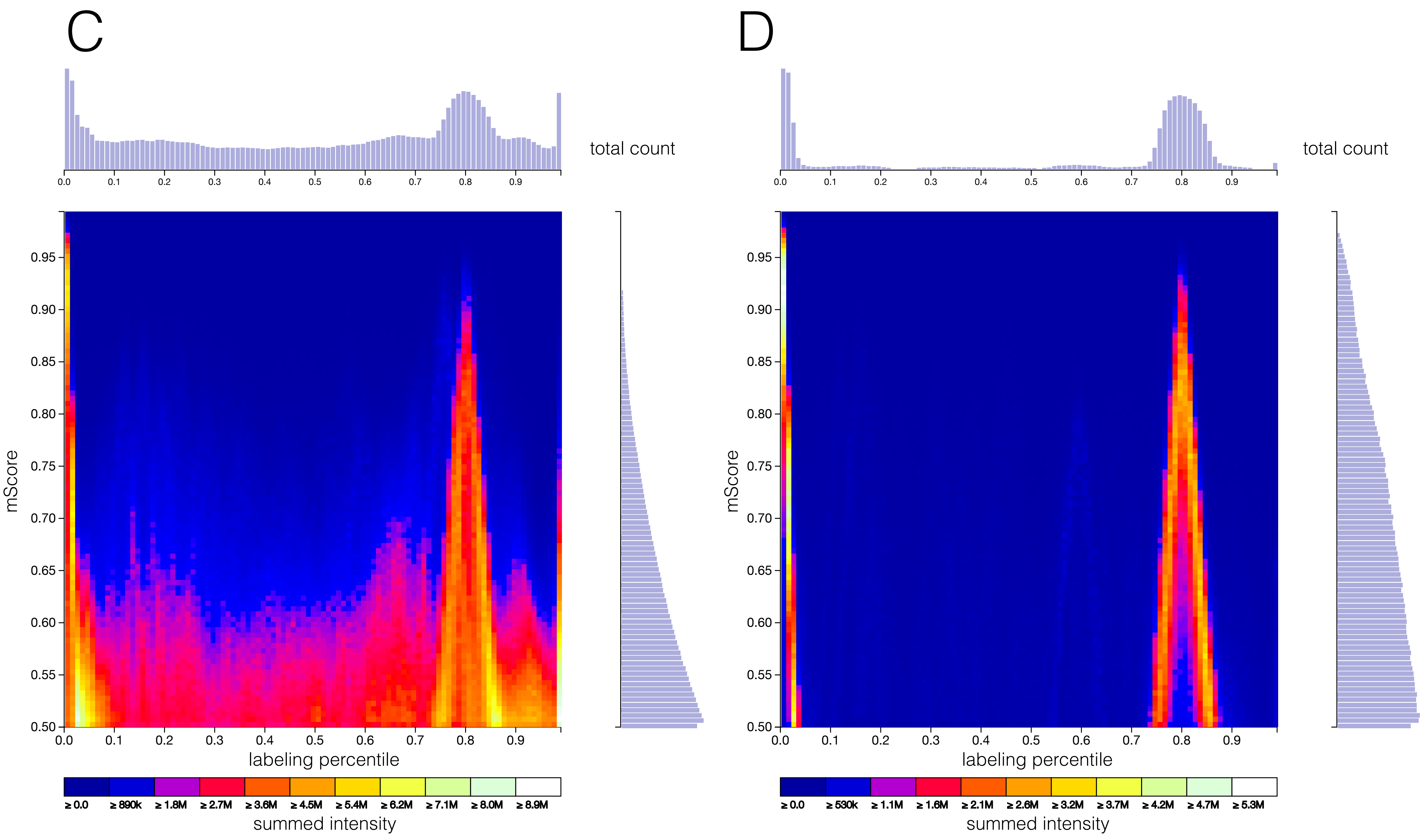

Heats maps for the 0 and 80% mixture of pulse chase gold standard data set. C, all peptides were quantified in all spectra. D, only quantifications of peptides were allowed for spectra which appeared in stringent determined peptide specific retention time windows.

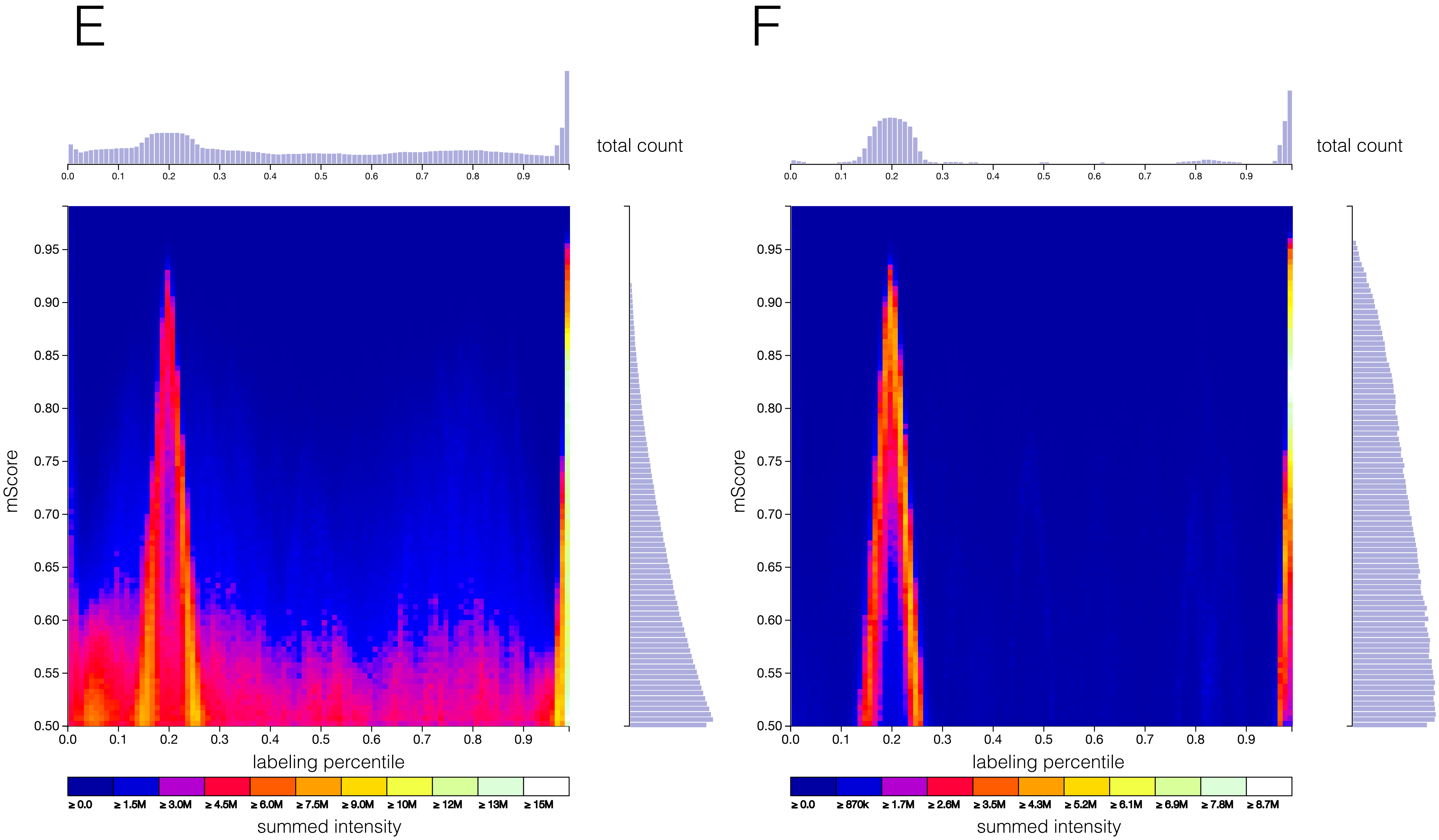

Heats maps for the 20 and 100% mixture of pulse chase gold standard data set. E, all peptides were quantified in all spectra. F, only quantifications of peptides were allowed for spectra which appeared in stringent determined peptide specific retention time windows.

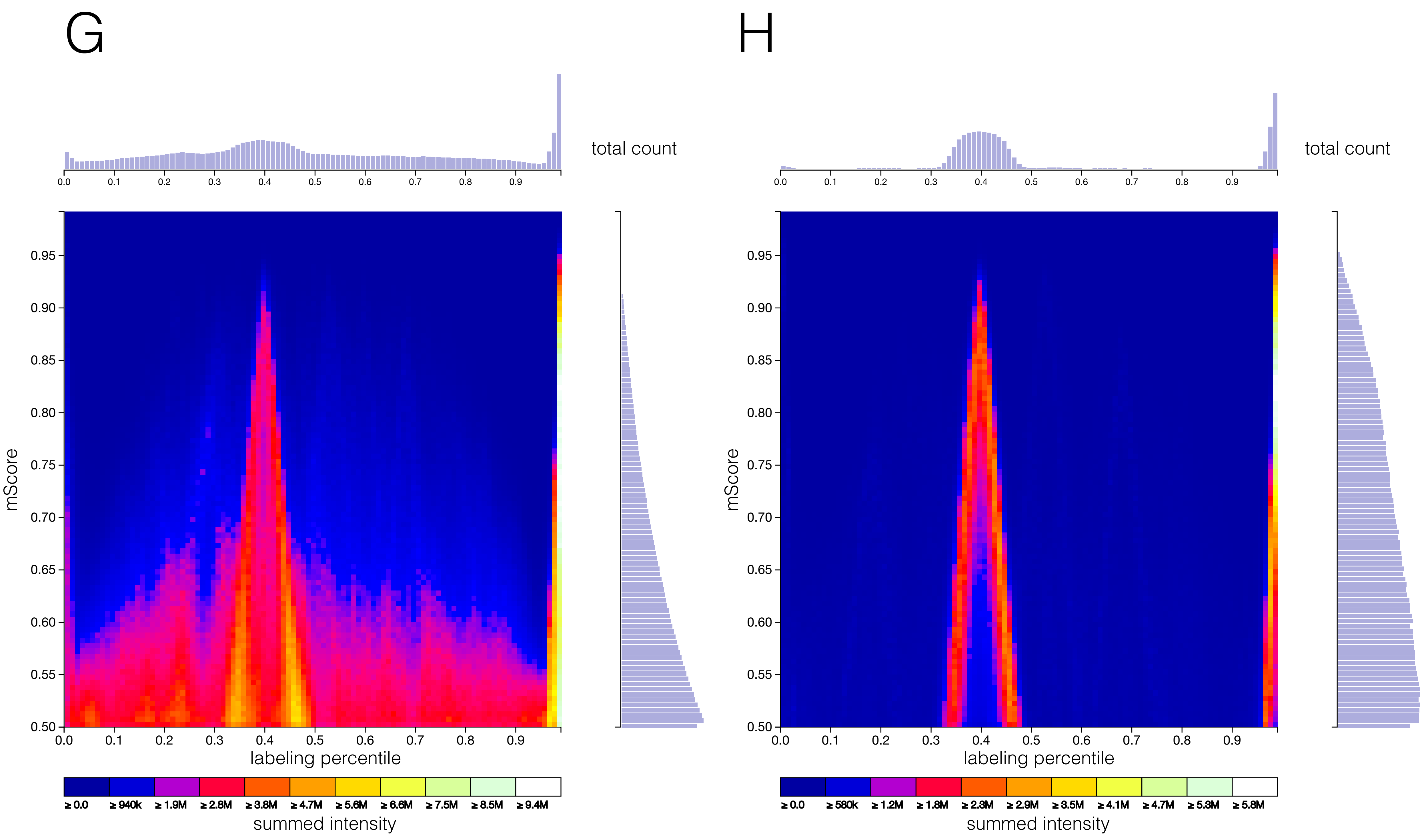

Heats maps for the 40 and 100% mixture of pulse chase gold standard data set. G, all peptides were quantified in all spectra. H, only quantifications of peptides were allowed for spectra which appeared in stringent determined peptide specific retention time windows.

Supplemental Figure 13

Retention-time window length distribution of pulse chase gold standard data set before and after retention time alignment

Retention-time window length distribution density plot. Shown is the frequency of the length of the retention-time windows in minutes for 19,976 peptides before (green) and after the alignment (red). Retention time windows are defined by the difference between the latest and the earliest identification (PEP <= 0.01) over all LC-MS/MS runs. Each RT window was extended by 1 min in both directions. RT windows were determined using piqDB as described earlier (Hoehner *et al.* 2012, Barth *et al.* 2014)

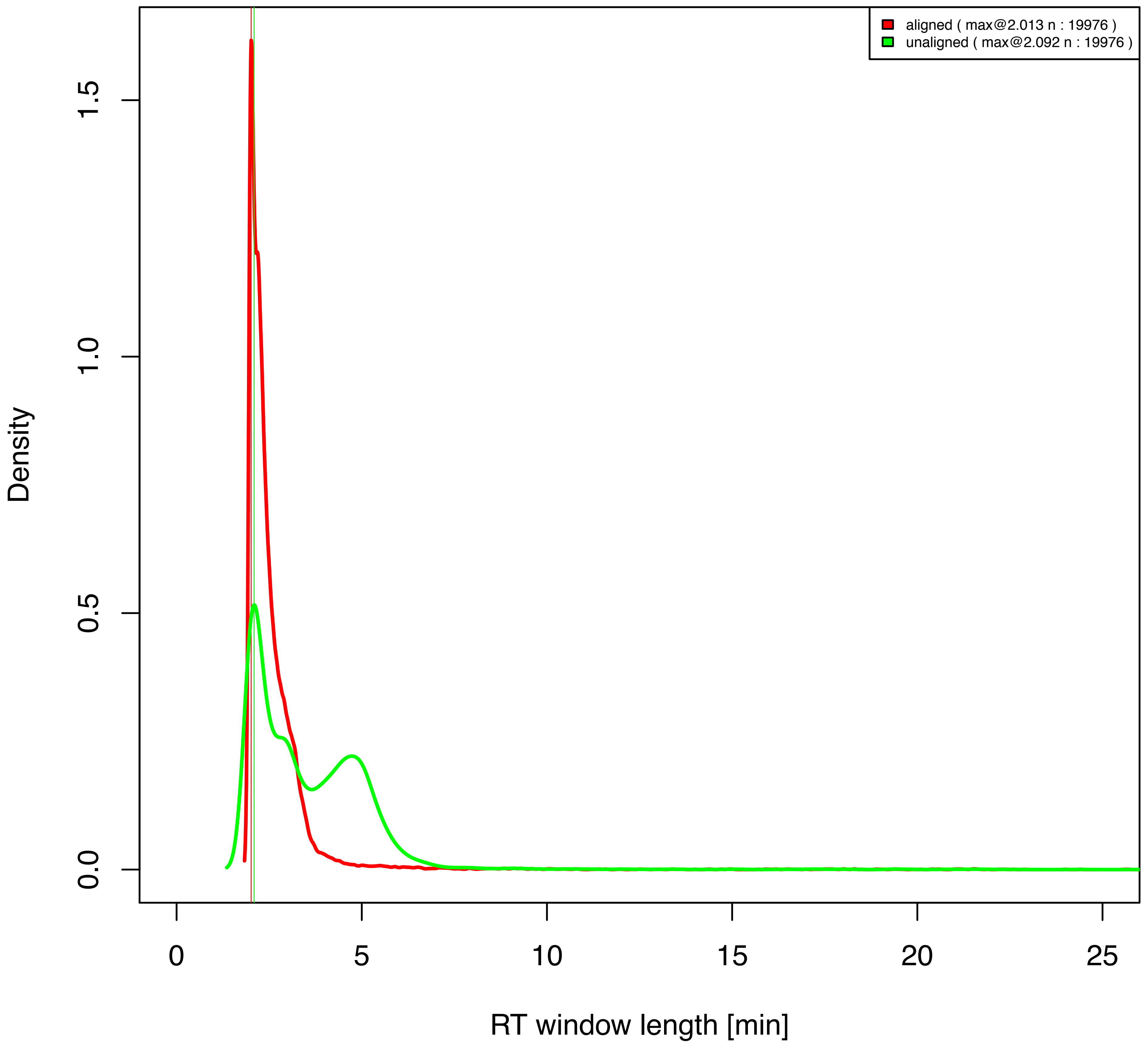
aligned (max@2.013 n : 19976)
unaligned (max@2.092 n : 19976)
Density
1.5
1.0
0.5
0.0
0
5
10
15
20
25
RT window length [min]

Supplemental Figure 14

Overlapping isotopologue examples.

Overlapping isotopologue examples of 0, 20, 40, 60, 80 and 100% enrichment of 99.4% enriched ^{15}N. The theoretical isotopologues of an example peptide are shown to illustrate the overlapping isotopic peaks at various positions of each isotopologue pattern. X-axis represents the m/z dimension, y-axis the relative intensity dimension. Isotopologues were colour coded to highlight the overlapping peaks: 0% cyan; 20% orange, 40% purple, 60% green, 80% light blue, 100% dark blue.

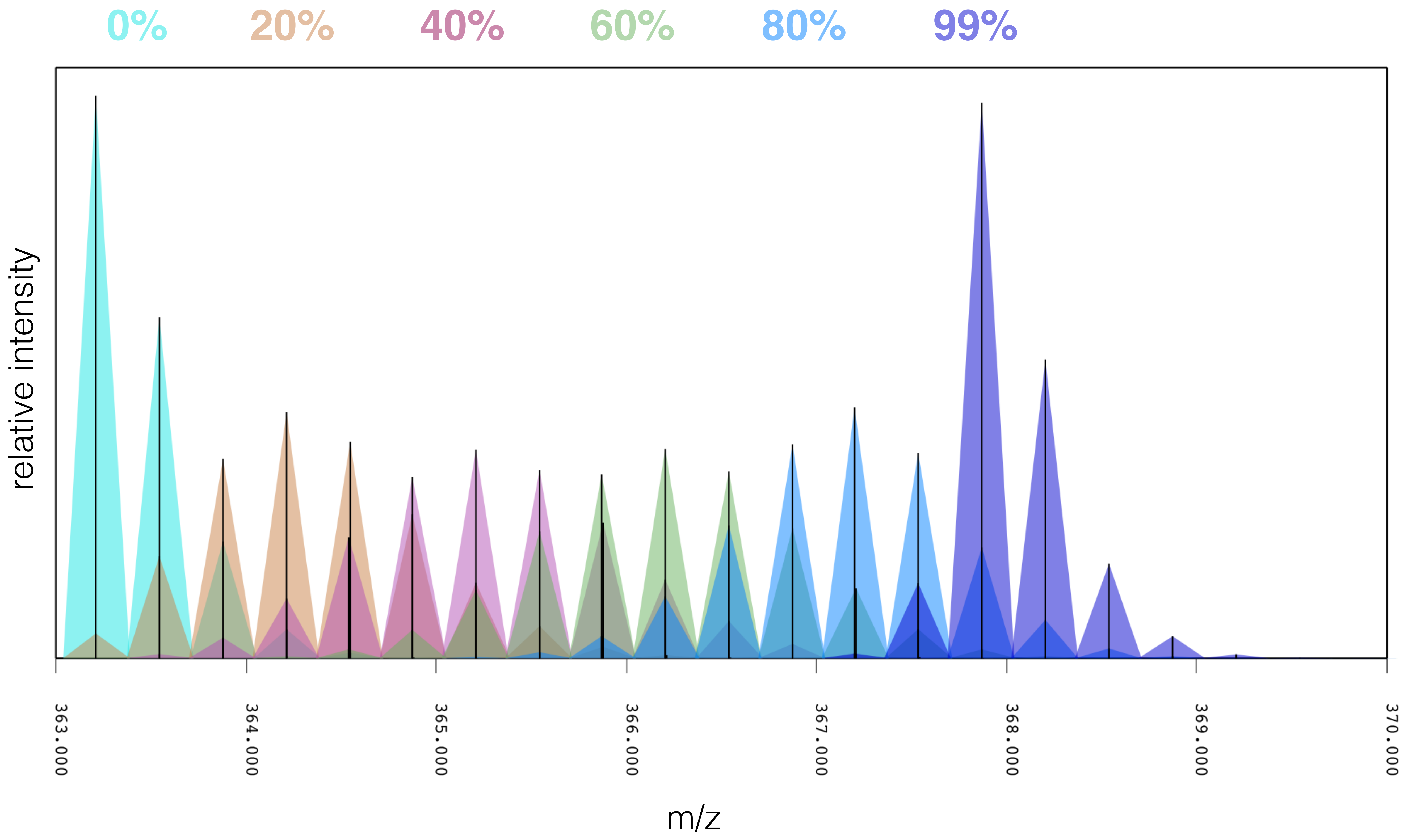

Isotopologues were colour coded to highlight the overlapping peaks: 0% cyan; 20% orange, 40% purple, 60% green, 80% light blue, 100% dark blue

Supplemental Figure 15

mScore dependent quantification accuracy - spiked-in proteins

The mScore dependent accuracy of pyQms. The absolute deviation of log2 peptide ratios from the ground truth was assessed for all 12 spiked-in proteins in the data set of Bruderer *et al.* (2015). The observed label-free log2 peptide ratio between all sample combinations was calculated. The deviation between the observed ratio and expected (ground truth) ratio (log2) was calculated and are displayed as density plot and box plots in the inset on the right. The mScore is defined as the average from both samples compared. The ratios were binned according to this average mScore. Density plot: x-axis, absolute deviation of log2 peptide ratios from log2 ground truth ratio; y-axis, density. Box plot: x-axis, mScore bin; y-axis, absolute deviation of log2 peptide ratios from log2 ground truth ratio. The legend shows the total number of peptide ratios compared (n) and the x-position of the maximum density (‘max@‘).

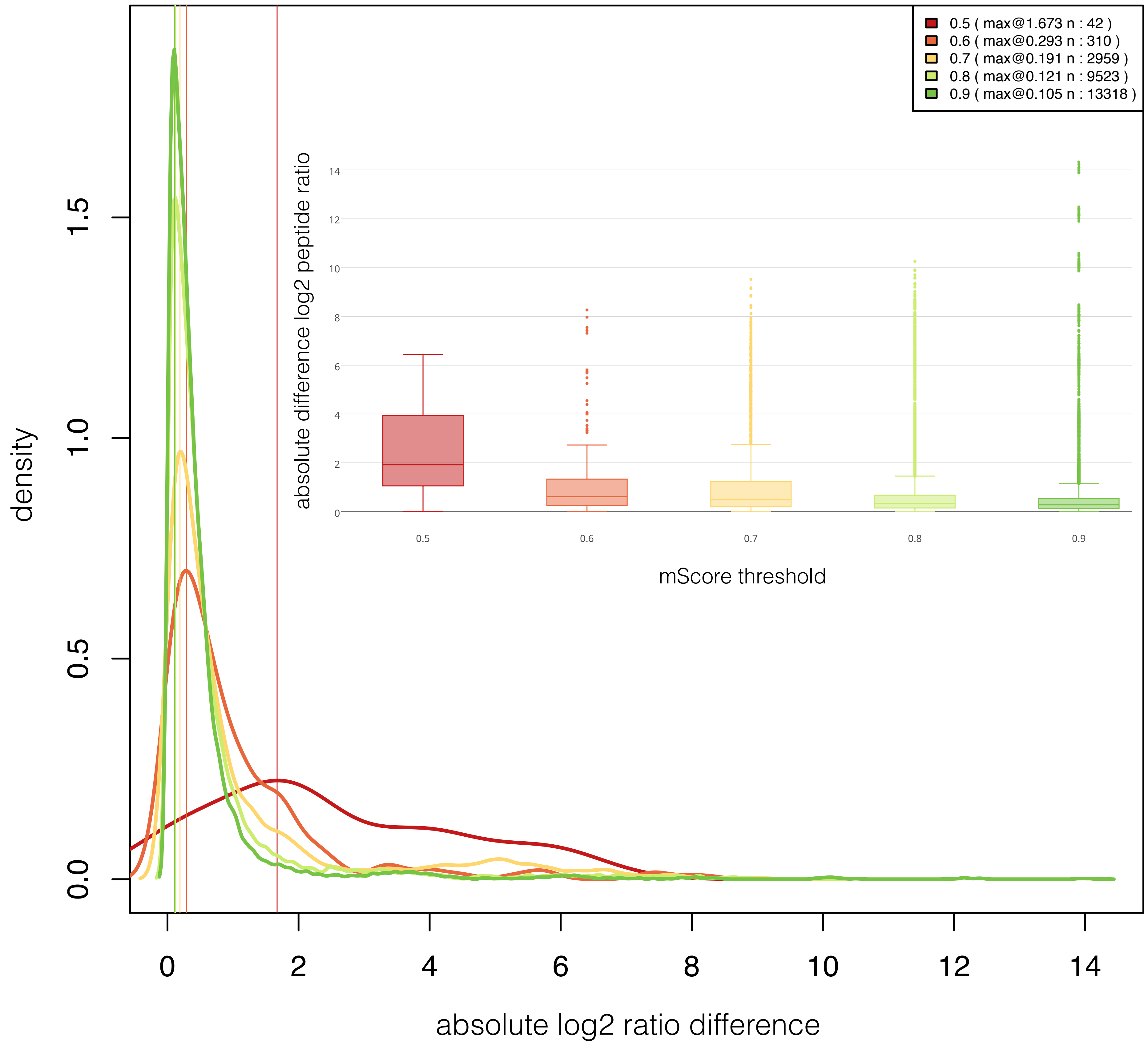

0.5 (max@1.673 n : 42)
0.6 (max@0.293 n : 310)
0.7 (max@0.191 n : 2959)
0.8 (max@0.121 n : 9523)
0.9 (max@0.105 n : 13318)
absolute difference log2 peptide ratio
mScore threshold
0.5
0.6
0.7
0.8
0.9
density
0.0
0.5
1.0
1.5
0
2
4
6
8
10
12
14
absolute log2 ratio difference
