# Supplementary material for: pyQms enables universal and accurate quantification of mass spectrometry data
Source: Mol Cell Proteomics. 2017 Jul 20;16(10):1736–45. doi: 10.1074/mcp.M117.068007 (PMC5629261; doi:10.1074/mcp.M117.068007)
Supplement: Supplemental Data [file 10.1074_M117.068007_mcp.M117.068007-3.pdf]

Supplemental methods

The terms metabolic labeling and fixed labeling

In general, metabolic labeling is equally referred to SILAC (1) and to any other stable isotope labeling method like ^{15}N (Fig. 1). This view comes with certain inconsistencies from a chemical perspective, which leads to inaccurate isotopologue calculations, especially when both methods are combined. A SILAC peptide labeled with heavy arginine ($C_6H_{12}{}^{15}N_4O$) contains two distinct nitrogen pools that show different isotopic distributions: the labeled Arg is enriched in ^{15}N, while the rest of the peptide shows the natural abundance of nitrogen. As a result, those two nitrogen pools have to be considered separately in order to calculate high precision isotopologues. Thus SILAC labeling induces a fixed mass shift similar to any chemical labeling, while metabolic labeling with ^{15}N changes the natural isotopic distribution to an artificial enriched percentile in all molecules, thereby changing the form of the isotopologue pattern. Therefore, we would like to propose a redefinition of metabolic labeling in a more consistent way, in which only labels that are metabolized and incorporated through metabolic pathways are labeled as metabolic labeling. While the isotopic labeled amino acids in a SILAC experiments are taken up by the cell, the label itself is not metabolized. In fact, proline is added additionally to minimize the dilution of the label all other pools / amino acids. Thus true metabolic labeling allows partial labeling with x %, while fixed labeling is describing an artificially attached molecule, be it *in vivo* (SILAC) or *in vitro* (chemical tagging). This convention is used in pyQms,

where metabolic labeling is used to be defined as differential label incorporation percentiles for given elements (e.g. ^{15}N enriched to x %) and fixed labeling is used to defined modification that do not change the shape of the isotopologue but introduce a given mass shift like SILAC, carbamidomethylation, ^{18}O digestion (2), iTRAQ (3), TMT (4) or any other chemical modification that are part of the community curated collection at unimod.org.

Other algorithms utilizing isotopologue information for peptide quantification in comparison to pyQms

Multiple available algorithms use the isotopologue information to improve the molecule detection for peptide quantifications (reviewed in (5–7)). Various approaches are employed to incorporate the isotopologue information. In the following section the approaches of the algorithms are summarized and finally compared to pyQms. Please refer also to supplemental Table S1 for an extended overview of algorithms for MS based molecule quantitation, to our current knowledge. Additionally, tools for metabolite quantitation and algorithms detecting partial labeled molecules are compared to pyQms. Please note that information in supplemental Table S1 is presented to the best of our knowledge. Nevertheless, we will make this table publicly available for comments and extensions under:

https://docs.google.com/spreadsheets/d/18_h3ACxCDjMynptcm9uU8E61RxE-o7r_qlVq2vT9xng/edit?usp=sharing

Algorithms

msInpsect (8) detects potential isotopic patterns and matches them to peptides predicting isotopologues using a Poisson distribution, which was empirically evaluated using human tryptic peptides. Only the most intense peak from the isotopologue is used for quantification, since it is argued that this peak is measured with the highest precision (8).

MapQuant (9) determines isotopic clusters considering ^{13}C (and its natural distribution) for the calculation of the isotopic pattern. The software uses a binomially distributed sum of 2-D Gaussians as a bivariate function of retention time and *m/z* to fit peaks which are potential isotopic clusters (9).

SpecArray (10) defines peptide features by their monoisotopic mass, charge and retention time at peak apex. Signals are compared to peptide isotopic distributions to identify potential peptide features. Signals from the first three calculated and found isotopic masses are combined for the peptide signal. The quality of the feature is defined as the single to noise ratio at the features apex and the abundance as the feature area.

OpenMS (11) uses the ‘averagine’ model to approximate the amino acid composition of a given mass and calculate atomic composition and isotope distributions. Spectra a filtered to find wavelet based isotopic patterns of peptides of a given charge state. Several extensions to OpenMS exists nowadays, which refine the feature detection and broaden the application range also to metabolomics and further research fields (12, 13).

Superhirn (14) detects the monoisotopic peak by comparing measured values to isotopic templates. Overlapping isotopologues can be resolved. The intensity is defined as the intensity of the complete isotopic pattern. Monoisotopic masses are clustered in retention time dimension (with a *m/z* tolerance of 0.005 Da) and the MS^1 feature is defined.

Census (15) uses the amino acid elemental composition to determine isotopologues. The composition can be altered so that any labeling strategy should be applicable. A *m/z* range is determined based on the isotopologue pattern and isotope ion intensities are extracted within this range (at least 5 % of isotopologue base peak intensity) using a *m/z* tolerance defined by the user.

MaxQuant (16) detects three dimensional peptide features and uses a graph model to assign these features to isotopologues of peptides. The 'averagine' model is used to define the charge dependent mass difference between isotopologue peaks. Isotope patterns are refined by splitting them according to obtain groups for single charge states and retaining the charge state with the highest number of peaks. Peptide intensity if defined as the two dimensional centroid intensity (sum of raw intensities) of an Isotope pattern (16).

Pyquant (17) detects isotope patterns and extracts XICs from raw data. Amounts are calculated from the area under the curve for the XICs employing a Bi-Gaussian fit. For validation several quality measures are applied, like

signal to noise ratio, intensity of the quantified molecule, as well as the elution peak width and density (17).

pySM (18) is a identification and quantification tool for metabolomics analyzed by imaging MS. In a first step MS images are filtered to separate for information and only noise containing ion images. pySM uses a scoring system (MSM score) to evaluate the match of the isotopic pattern in an approach comparable to pyQms. Prior to matching of the isotope envelope, spectra are averaged, where the principal peak of the isotope pattern is present. The main aim of pySM is to identify metabolites using a FDR approach by assuming implausible adducts to generate decoy molecules (18).

Differentiation of pyQms to other algorithms

In contrast to other algorithms pyQms does not determine peptide features, or in a more general respect molecule features, but takes a given input set of molecules and matches the calculated isotope pattern in every given MS spectra. pyQms is optimized in such a way that such a computationally costly approach can nevertheless be taken. User defined filtering steps and elution profile reconstructions are done after the raw quantification procedure. According to the different needs of analyses this filtering can be very diverse. Therefore, pyQms was designed to perform the raw quantification task in the analyses and further result integration is left to dedicated, specialized tools, algorithms and/or pipeline frameworks. However, the pyQms offers basic functionality that allows users to tag elution profile peaks with given identities, e.g. peptide search results from Ursgal (19) can be parsed and used to define retention time windows. Equally input files for metabolomics data can be

created by the user, defining e.g. trivial names and retention time windows. Please refer to the documentation for more details.

In pyQms, molecules are quantified on spectrum level by scaling the theoretical isotope pattern to the measured ions. This scaling represents the amount of this molecule in this spectrum, because the sum of all theoretical isotope pattern peaks are equal to 1. Other tools tend to use only the most abundant peak (very often the monoisotopic peak, which is very frequently not the most abundant peak when larger peptides are considered (7)), only use a part of the isotopologue for amount determination or uses the average of multiple spectra. Thus, selecting the monoisotopic peak is in general very difficult, especially if elemental enrichment levels are between 20 to 80%; see e.g. supplemental Fig. 14.

The assembly of features is not implemented in pyQms as it is a raw quantification tool. Nevertheless isotopologue matches can be assembled in time dimension (with defined RT borders e.g. obtained by peptide identifications), which yields an accurate representative value for a MS run. Current default behavior is to extract the maximum intensity, the sum of all spectra and the area under curve of a molecular formula from one MS run. Nevertheless, newly innovative algorithms can also be easily incorporated into pyQms (please refer to online documentation). Also evidence files (with peptide identification information) can be provided and this information is subsequently used to define retention time windows and calculate amounts.

Further pyQms uses no approximations like the ‘averagine’ model or uses common approximate isotopic pattern, but incorporates all elemental isotope abundances into the calculation of the isotopologues, thereby increasing the precision of the quantification. This is especially important considering the fact that instruments become more and more precise. Further, highly accurate isotopologue calculation in combination with high precision instruments will pave the way to identification free peptide quantification.

Generally, quantitative MS analyses will benefit from the mScore. More accurate label-free amount and subsequent ratio determination between complex samples are obtained if the mScore thresholds are increased (supplemental Fig. 15).

References:

1. Ong, S.-E. (2002) Stable Isotope Labeling by Amino Acids in Cell Culture, SILAC, as a Simple and Accurate Approach to Expression Proteomics. *Mol. Cell. Proteomics* 1, 376–386

2. Yao, X., Freas, a., Ramirez, J., Demirev, P. a., and Fenselau, C. (2001) Proteolytic 18O labeling for comparative proteomics: Model studies with two serotypes of adenovirus. *Anal. Chem.* 73, 2836–2842

3. Wiese, S., Reidegeld, K. a, Meyer, H. E., and Warscheid, B. (2007) Protein labeling by iTRAQ: a new tool for quantitative mass spectrometry in proteome research. *Proteomics* 7, 340–50

4. Thompson, A., Schäfer, J., Kuhn, K., Kienle, S., Schwarz, J., Schmidt, G., Neumann, T., Johnstone, R., Mohammed, a K. a, and Hamon, C. (2003) Tandem mass tags: a novel quantification strategy for comparative analysis of complex protein mixtures by MS/MS. *Anal. Chem.* 75, 1895–904

5. Gonzalez-Galarza, F. F., Lawless, C., Hubbard, S. J., Fan, J., Bessant, C., Hermjakob, H., and Jones, A. R. (2012) A critical appraisal of techniques, software packages, and standards for quantitative proteomic analysis. *OMICS* 16, 431–442

6. Nahnsen, S., Bielow, C., Reinert, K., and Kohlbacher, O. (2013) Tools for label-free peptide quantification. *Mol. Cell. Proteomics* 12, 549–56

7. Sandin, M., Teleman, J., Malmström, J., Levander, F., Stephan, C., and All, E. B. V (2014) Biochimica et Biophysica Acta Data processing

methods and quality control strategies for label-free LC – MS protein quantification ✩. *BBA - Proteins Proteomics* 1844, 29–41

8. Bellew, M., Coram, M., Fitzgibbon, M., Igra, M., Randolph, T., Wang, P., May, D., Eng, J., Fang, R., Lin, C., Chen, J., Goodlett, D., Whiteaker, J., Paulovich, A., and McIntosh, M. (2006) A suite of algorithms for the comprehensive analysis of complex protein mixtures using high-resolution LC-MS. *Bioinformatics* 22, 1902–1909

9. Leptos, K. C., Sarracino, D. a., Jaffe, J. D., Krastins, B., and Church, G. M. (2006) MapQuant: Open-source software for large-scale protein quantification. *Proteomics* 6, 1770–1782

10. Li, X., Yi, E. C., Kemp, C. J., Zhang, H., and Aebersold, R. (2005) A software suite for the generation and comparison of peptide arrays from sets of data collected by liquid chromatography-mass spectrometry. *Mol. Cell. Proteomics* 4, 1328–1340

11. Sturm, M., Bertsch, A., Gröpl, C., Hildebrandt, A., Hussong, R., Lange, E., Pfeifer, N., Schulz-Trieglaff, O., Zerck, A., Reinert, K., and Kohlbacher, O. (2008) OpenMS – An open-source software framework for mass spectrometry. *BMC Bioinformatics* 9, 163

12. Kenar, E., Franken, H., Forcisi, S., Wörmann, K., Häring, H.-U., Lehmann, R., Schmitt-Kopplin, P., Zell, A., and Kohlbacher, O. (2014) Automated label-free quantification of metabolites from liquid chromatography-mass spectrometry data. *Mol. Cell. Proteomics* 13, 348–59

13. Sachsenberg, T., Herbst, F.-A., Taubert, M., Kermer, R., Jehmlich, N., Bergen, M. Von, Seifert, J., and Kohlbacher, O. (2015) MetaProSIP: Automated Inference of Stable Isotope Incorporation Rates in Proteins for Functional Metaproteomics. *J. Proteome Res.* 14, 619–627

14. Mueller, L. N., Rinner, O., Schmidt, A., Letarte, S., Bodenmiller, B., Brusniak, M.-Y., Vitek, O., Aebersold, R., and Müller, M. (2007) SuperHirn – a novel tool for high resolution LC-MS-based peptide/protein profiling. *Proteomics* 7, 3470–3480

15. Park, S. K., Venable, J. D., Xu, T., and Yates, J. R. (2008) A quantitative analysis software tool for mass spectrometry–based proteomics. *Nat. Methods* 5, 319–322

16. Cox, J., and Mann, M. (2008) MaxQuant enables high peptide identification rates, individualized p.p.b.-range mass accuracies and proteome-wide protein quantification. *Nat. Biotechnol.* 26, 1367–1372

17. Mitchell, C. J., Kim, M.-S., Na, C. H., and Pandey, A. (2016) PyQuant: A Versatile Framework for Analysis of Quantitative Mass Spectrometry Data. *Mol. Cell. Proteomics* 15, 2829–2838

18. Palmer, A., Phapale, P., Chernyavsky, I., Lavigne, R., Fay, D., Tarasov, A., Kovalev, V., Fuchser, J., Nikolenko, S., Pineau, C., Becker, M., and Alexandrov, T. (2016) FDR-controlled metabolite annotation for high-resolution imaging mass spectrometry. *Nat. Methods* 14, 1–8

19. Kremer, L. P. M., Leufken, J., Oyunchimeg, P., Schulze, S., and Fufezan, C. (2016) Ursgal, Universal Python Module Combining

Common Bottom-Up Proteomics Tools for Large-Scale Analysis. *J. Proteome Res.* 15, 788–794
