# Supplementary material for: pyQms enables universal and accurate quantification of mass spectrometry data
Source: Mol Cell Proteomics. 2017 Jul 20;16(10):1736–45. doi: 10.1074/mcp.M117.068007 (PMC5629261; doi:10.1074/mcp.M117.068007)
Supplement: Supplemental Data [file 10.1074_M117.068007_mcp.M117.068007-4.pdf]

Supplemental Table S1

Overview of quantification engines for DDA and DIA mass spectrometry data. The table covers approaches for metabolic (including partial labeling), chemical labeling as well as label free approaches in metabolomics and proteomics as main research fields. The ability of each software to perform the labeling strategies in Fig. 1 are evaluated. These labeling strategies are further specified. The possibility of analyzing DDA and DIA data are described. Finally matching and accessibility properties are addressed. The table was generated to the best of our knowledge. The table is publicly available and open for comments and extensions.

(https://docs.google.com/spreadsheets/d/18_h3ACxCDjMynptcm9uU8E61RxE-o7r_qlVq2vT9xng/edit?usp=sharing)

Supplemental Table S2

Reports all identified peptides ('Peptide') (PEP <=1%) in the pulse gold standard data set. The proteins identifiers ('Protein') are reported as well. Any deviation from the expected trypsin cleavage is indicated ('Cleaved_Specifically'; 'True', peptide is trypsin specific; 'False', peptide is not trypsin specific). All identification scores as posterior error probabilities ('PEPs', separated by ';') are reported. Furthermore, the m/z for the ^{14}N ('m/z 14N') and ^{15}N ('m/z 15N') version are given if identified in the respective labeling state.

Supplemental Table S3

For every protein ('Protein') the number of distinct unique identified peptides ('Number_Peptides') as well as the sequence coverage ('Sequence_Coverage [%]') of the protein are shown. Further all peptides identified for the protein are reported ('Peptides', individual peptides separated by ';').

Supplemental Table S4

Reports all retention time windows. For each peptide ('Peptide') and the according molecular formula ('Formula') the start and stop of the aligned ('RT window aligned start [min]', 'RT window aligned stop [min]') and the unaligned ('RT window unaligned start [min]', 'RT window unaligned stop [min]') RT window is reported in minutes. Please note that for quantification a retention time border tolerance of +/- 1 min was applied.

Supplemental Table S5

Shows the retention time alignment functions for each DDA sample/LC-MS/MS run from the Bruderer *et al.* (2015) data set determined by piqDB. The original retention time is reported ('Retention time source [min]'). The target retention times are reported for every sample/LC-

MS/MS run ('B_D140314_SGSDSsample[x]_R0[y]_MSG_T0', with [x] referring to sample number and [y] referring to replicate number). Please note that the same functions were used for the DIA samples/LC-MS/MS runs ('B_D140314_SGSDSsample[x]_R0[y]_MHRM_T0'), since they were measured directly after their corresponding DDA samples.

Supplemental Table S6

Reports the intensity correction factors ('alignment factor') for each sample/LC-MS/MS run ('Filename/MS run') determined by piqDB. All samples/LC-MS/MS runs were aligned to sample 'B_D140314_SGSDSsample1_R01_MSG_T0' ('Normalized to MS run')

Supplemental Table S7

Holds the DDA quantification results for the spiked-in-proteins of the DDA data set from Bruderer *et al.* (2015). All quantified peptides ('Peptide') in the according charge state ('Charge') with their corresponding protein ('Protein') are shown. The maximum intensity of the peptide in the retention time window is shown for each sample. Descriptions of the column headers ('S[x]R[y]', with x referring to sample number and y referring to replicate number) with reference to the sample/LC-MS/MS run can be found in supplemental table S12.

Supplemental Table S8

Contains the complete curated spectral library used for the DIA quantification approach with pyQms. For each peptide ('Peptide') and the corresponding protein ('Protein') the fragment or parent ion name including charge ('Ion Name') and the respective molecular formula ('Ion formula') is shown. Usage of each ion for the peptide intensities in each sample is also reported ('Sample').

Supplemental Table S9

Holds the same information as supplemental stable S7 but for the DIA quantification results. Peptide intensities are sums of fragment ion intensities summed up on peptide level. Descriptions of the column headers ('S[x]R[y]', with [x] referring to sample number and [y] referring to replicate number) with reference to the sample/LC-MS/MS run can be found in supplemental Table S12.

Supplemental Table S10

Shows the summarized t-test results on master mix level for the DDA and DIA approaches by pyQms ('MM[x]_pyqms_dda', 'MM[x]_pyqms_dia'), Spectronaut ('MM[x]_bruderer_hrm') and MaxQuant ('MM[x]_bruderer_dda') ('Analysis identifier', [x] indicates the analyzed master mix). Relative occurrences of the p-values of all 12 spiked-in-proteins on master mix level are

shown. Calculated p-values for peptide ratios on protein level were counted for DDA and DIA mode separately and for amounts determined by pyQms (for DDA and DIA) as well as MaxQuant (DDA) and Spectronaut (DIA). Missing ratios were omitted. The level of significance is indicated as not significant different (‘nsd’) or by p-value threshold (‘*’, p-value ≤0.05; ‘**’, p-value ≤0.01; ‘***’, p-value ≤0.001).

Supplemental Table S11

Shows the summarized t-test results on protein level for the DDA and DIA approaches by pyQms (‘MM[x]_[y]_pyqms_dda’, ‘MM[x]_[y]_pyqms_dia’), Spectronaut (‘MM[x]_[y]_bruderer_hrm’) and MaxQuant (‘MM[x]_[y]_bruderer_dda’) ('Analysis identifier’, [x] indicates the analyzed master mix, [y] indicates the tested protein identifier). Relative occurrences of the p-values of all 12 spiked-in-proteins on master mix level are shown. Calculated p-values for peptide ratios on protein level were counted for DDA and DIA mode separately and for amounts determined by pyQms (for DDA and DIA) as well as MaxQuant (DDA) and Spectronaut (DIA). Missing ratios were omitted. The level of significance is indicated as not significant different (‘nsd’) or by p-value threshold (‘*’, p-value ≤0.05; ‘**’, p-value ≤0.01; ‘***’, p-value ≤0.001).

Supplemental Table S12

Header/sample descriptions for the supplemental table headers. The header column indicates the referenced sample or LC-MS/MS run in the data set of Bruderer *et al.* (2015). Please note that ‘MSG’ in sample/LC-MS/MS run names refers to DDA measuring mode (for table S2) and ‘MHRM’ refers to DIA measuring mode (for supplemental Table S9 und S8).
